# Supplementary material for: Synthesis and Evaluation of 11-Butyl Matrine Derivatives as Potential Anti-Virus Agents
Source: Molecules. 2022 Nov 4;27(21):7563. doi: 10.3390/molecules27217563 (PMC9658933; doi:10.3390/molecules27217563)
Supplement: Supplementary file 1 [file molecules-27-07563-s001.zip › molecules-1993088-supplementary.pdf]

Synthesis and evaluation of 11-butyl matrine derivatives as potential anti-virus agents

Wanjun Ni, Lizhong Wang, Hongjian Song, Yuxiu Liu,* and Qingmin Wang*

State Key Laboratory of Elemento-Organic Chemistry; Research Institute of Elemento-Organic Chemistry, College of Chemistry, Nankai University; Frontiers Science Center for New Organic Matter; Tianjin 300071, People's Republic of China

(Supporting Information)

Tables of Content

1. **Data for compounds 1-21……………………………………………………………….…...2**

2. **^{1}H NMR and ^{13}C NMR spectra for compounds B-F, 1-21……………………………..…11**

3. **Detailed bioassay procedures …………………………………………………………..…..34**

4. **Insecticidal activity (Table S1) ……………………………………………….….….…..37**

5. **Fungicidal activity (Table S2) ……………………………………………………....…..38**

1. Data for compounds 1-21

N-cyclopropyl sulfonyl-11-butyl matrine derivative (**1**)

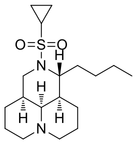

Yellow oil 0.3 g, yield 53%. ^{1}H NMR (400 MHz, $CDCl_3$) δ 3.72 (dd, *J* = 12.0, 8.0 Hz, 1H), 3.42 (d, *J* = 8.0 Hz, 2H), 2.77 (t, *J* = 12.0 Hz, 2H), 2.50 – 2.44 (m, 1H), 2.10 – 1.17 (m, 22H), 1.02 – 0.96 (m, 1H), 0.92 (t, *J* = 8.0 Hz, 3H). ^{13}C NMR (100 MHz, $CDCl_3$) δ 63.5, 57.9, 56.9, 47.7, 39.7, 34.7, 31.3, 30.1, 28.3, 28.0, 27.9, 22.9, 21.0, 20.9, 14.1, 5.9, 5.1. HRMS (ESI) calcd for $[C_{18}H_{32}N_2O_2S+H]^+$ 341.2257, found 341.2260.

N-methanesulfonyl-11-butyl matrine derivative (**2**)

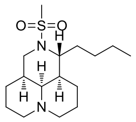

White solid 0.30 g, yield 75%. Mp 78–79 °C. ^{1}H NMR (400 MHz, $CDCl_3$) δ 3.61 (d, *J* = 4.0 Hz, 1H), 3.44 – 3.39 (m, 1H), 3.29 (t, *J* = 12.0 Hz, 1H), 2.91 (s, 3H), 2.74 (s, 2H), 2.05 – 1.73 (m, 9H), 1.59 – 1.26 (m, 10H), 0.92 (t, *J* = 4.0 Hz, 3H). ^{13}C NMR (100 MHz, $CDCl_3$) δ 63.1, 57.3, 56.8, 56.7, 46.3, 39.7, 39.0, 34.2, 32.2, 28.4, 27.9, 27.7, 21.1, 20.9. HRMS (ESI) calcd for $[C_{16}H_{30}N_2O_2S+H]^+$ 315.2101, found 315.2190.

N-(3-pyridinesulfonyl)-11-butyl matrine derivative (**3**)

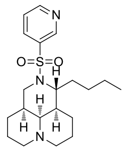

Yellow oil 0.60 g, yield 76%. ^{1}H NMR (400 MHz, $CDCl_3$) δ 9.07 (s, 1H, Py-H), 8.74 (d, *J* = 4.0 Hz, 1H), 8.14 (d, *J* = 8.0 Hz, 1H), 7.41 (dd, *J* = 8.0, 4.0 Hz, 1H), 3.66 (dd, *J* = 8.0, 4.0 Hz, 1H), 3.53 (dd, *J* = 8.0, 4.0 Hz, 1H), 3.31 – 3.26 (m, 1H), 2.56 (dd, *J* = 12.0 Hz, 1H), 2.48 (dd, *J* = 12.0 Hz, 1H), 1.98 (s, 2H), 1.79 – 1.71 (m, 6H), 1.50 (t, *J* = 12.0 Hz, 1H), 1.44 – 1.18 (m, 10H), 0.82 (t, *J* = 8.0 Hz, 3H). ^{13}C NMR (100 MHz, $CDCl_3$) δ 152.3, 148.5, 137.4, 135.1, 123.0, 62.61, 57.6, 56.4,

56.3, 45.9, 39.7, 34.1, 32.8, 28.7,28.2, 27.9, 22.7, 20.8, 20.4, 14.0. HRMS (ESI) calcd for $[C_{20}H_{31}N_3O_2S+H]^+$ 378.2210, found 378.2219.

N-vinylsulfonyl-11-butyl matrine derivative (**4**)

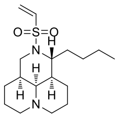

Yellow oil 0.25 g, yield 65%. ^1H NMR (400 MHz, $CDCl_3$) δ 6.55 (dd, J = 16.0, 8.0 Hz, 1H), 6.19 (d, J = 16.0 Hz, 1H), 5.86 (d, J = 12.0 Hz, 1H), 3.62 (dd, J = 8.0, 4.0 Hz, 1H), 3.38 (dd, J = 8.0, 4.0 Hz, 1H), 3.30 – 3.24 (m, 2H), 2.73 (t, J = 8.0 Hz, 1H), 2.05 (s, 1H), 1.97 (d, J = 4.0 Hz, 2H), 1.79 – 1.68 (m, 5H), 1.66 – 1.26 (m, 11H), 0.90 (t, J = 8.0 Hz, 3H). ^{13}C NMR (100 MHz, $CDCl_3$) δ 136.5, 124.7, 63.1, 57.5, 56.78, 56.70, 46.2, 39.8, 34.4, 31.8, 28.4, 27.9, 27.9, 22.7, 20.9, 20.7, 14.1. HRMS (ESI) calcd for $[C_{17}H_{30}N_2O_2S+H]^+$ 327.2101, found 327.2096.

N- benzenesulfonyl-11-butyl matrine derivative (**5**)

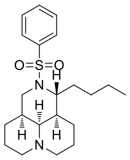

Yellow oil 0.43 g, yield 72%. ^1H NMR (400 MHz, $CDCl_3$) δ 7.85 (d, J = 8.0 Hz, 2H), 7.54 – 7.44 (m, 3H), 3.63 – 3.55 (m, 2H), 3.33 (t, J = 12.0 Hz, 1H), 2.63 (dd, J = 12.0, 8.0 Hz, 2H), 2.04 (d, J = 12.0 Hz, 2H), 1.89 – 1.72 (m, 6H), 1.55 – 1.09 (m, 10H), 1.11 (t, J = 8.0 Hz, 1H), 0.76 (t, J = 8.0 Hz, 3H). ^{13}C NMR (100 MHz, $CDCl_3$) δ 141.4, 131.9, 128.5, 127.3, 63.3, 58.3, 56.8, 47.6, 39.6, 34.9, 31.0, 29.7, 29.7, 29.3, 28.3, 27.9, 22.7, 20.8, 13.9. HRMS (ESI) calcd for $[C_{21}H_{32}N_2O_2S+H]^+$ 377.2257, found 377.2261.

N-(2-fluorobenzoyl)-11-butyl matrine derivative (**6**)

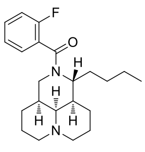

Yellow oil 0.64 g, yield 85%. ^1H NMR (400 MHz, $CDCl_3$) δ 7.39 (d, J = 8.0 Hz, 2H), 7.18 (t, J = 8.0 Hz, 1H), 7.09 (t, J = 8.0 Hz, 1H), 4.33 (s, 1H), 3.85 (s, 1H), 4.08 – 3.75 (m, 3H), 2.56 – 2.36 (m, 4H), 2.05 – 1.97 (m, 4H), 1.74

– 1.15 (m, 11H), 0.90 (t, *J* = 8.0 Hz, 3H). ^{13}C NMR (100 MHz, $CDCl_3$) δ 167.8, 132.8, 132.2, 131.4, 128.9, 125.3, 124.5, 123.6, 116.6, 116.1, 115.9, 99.9, 64.6, 60.3, 56.1, 50.2, 38.2, 36.9, 29.6, 28.3, 26.1, 22.7, 21.0, 19.3, 14.17,14.11. HRMS (ESI) calcd for $[C_{22}H_{31}FN_2O+H]^+$ 359.2493, found 359.2497.

N-benzoyl-11-butyl matrine derivative (**7**)

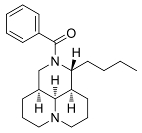

Yellow oil 0.30 g, yield 68%, ^{1}H NMR (400 MHz, $CDCl_3$) δ 7.43 (s, 2H), 7.37 (s, 3H), 4.01 (s, 1H), 3.75 (s, 1H), 3.43 – 3.08 (m, 1H), 2.76 (dd, *J* = 12.0, 4.0 Hz, 2H), 2.04 – 1.26 (m, 19H), 0.88 (t, *J* = 4.0 Hz, 3H).^{13}C NMR (100 MHz, $CDCl_3$) δ 171.6, 138.0, 67.9, 62.9, 56.7, 56.5, 54.6, 40.0, 35.2, 32.4, 28.7, 28.6, 22.7, 21.2, 20.9, 14.1.HRMS (ESI) calcd for $[C_{22}H_{31}N_2O+H]^+$ 341.2587, found 341.2593.

N-(1-naphthoyl)-11-butyl matrine derivative (**8**)

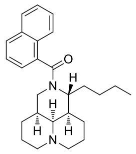

yellow oil 1.20 g, yield 75%. ^{1}H NMR (400 MHz, $CDCl_3$) δ 8.16 – 7.82 (m, 3H), 7.61 – 7.36 (m, 4H), 4.58 (s, 1H), 3.19 (s, 2H), 2.87 – 2.80 (m, 1H), 2.56 (s, 1H), 2.28 (s, 1H), 2.16 – 1.77 (m, 7H), 1.66 – 0.88 (m, 14H). ^{13}C NMR (100 MHz, $CDCl_3$) δ 171.3, 135.8, 133.5, 133.3, 130.1, 129.0, 128.4, 128.0, 126.3, 125.0, 124.2, 63.2, 55.9, 54.3, 47.7, 39.4, 36.0, 31.5, 28.7, 27.7, 26.6, 22.6, 21.6, 20.3, 19.6, 14.1. HRMS (ESI) calcd for $[C_{26}H_{34}N_2O+H]^+$ 391.2744, found 391.2747.

N-phenylacetyl-11-butyl matrine derivative (**9**)

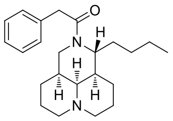

Yellow oil 0.45 g, yield 75%. ^{1}H NMR (400 MHz, $CDCl_3$) δ 7.29 – 7.22 (m, 5H), 4.13 – 4.06 (m, 1H), 3.75 – 3.46 (m, 4H), 3.00 (s, 2H), 2.02 – 1.28 (m, 19H), 0.87 (t, *J* = 4.0 Hz, 3H).^{13}C NMR (100 MHz, $CDCl_3$) δ 171.4,

135.5, 129.3, 128.9, 128.4, 126.5, 63.1, 56.3, 56.1, 55.1, 46.6, 42.6, 41.4, 38.8, 35.3, 32.0, 29.7, 28.6, 26.7, 22.8, 20.0, 14.1. HRMS (ESI) calcd for $[C_{23}H_{34}N_2O+H]^+$ 355.2744, found 355.2740.

N-(4-methylbenzoyl)-11-butyl matrine derivative (**10**)

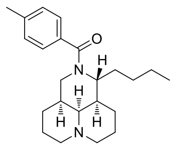

Yellow oil 0.51 g, yield 85%. ^{1}H NMR (400 MHz, $CDCl_3$) δ 7.37 (d, J = 8.0 Hz, 2H), 7.18 (d, J = 8.0 Hz, 2H), 4.05 (s, 1H), 3.83 (s, 1H), 3.39 (s, 1H), 2.98 (s, 2H), 2.37 (s, 4H), 2.08 – 1.80 (m, 8H), 1.50 – 1.26 (m, 10H), 0.88 (s, 3H). ^{13}C NMR (100 MHz, $CDCl_3$) δ 172.7, 139.7, 134.4, 129.5, 128.9, 127.3, 64.7, 63.8, 56.5, 56.4, 55.3, 38.9, 36.0,30.8, 28.5, 27.5, 22.9, 22.7, 21.6, 21.4, 20.3, 14.1. HRMS (ESI) calcd for $[C_{23}H_{34}N_2O+H]^+$ 355.2744, found 355.2752.

N-acryloyl-11-butyl matrine derivative (**11**)

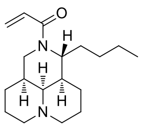

Yellow oil 0.52 g, yield 60%. ^{1}H NMR (400 MHz, $CDCl_3$) δ 6.63 (dd, J = 16.0, 12.0 Hz, 1H), 6.19 (d, J = 16.0 Hz, 1H), 5.58 (d, J = 12.0 Hz, 1H), 4.15 – 3.84 (m, 2H), 3.14 (s, 1H), 2.67 (s, 2H), 1.83 (s, 1H), 1.70 – 1.09 (m, 18H), 0.88 (t, J = 8.0 Hz, 3H). ^{13}C NMR (100 MHz, $CDCl_3$) δ 166.7, 129.6, 125.8, 62.4, 56.5, 56.3, 54.7, 40.2, 39.9, 34.4, 33.3, 29.1, 28.8, 28.5, 22.7, 21.3, 20.9, 14.0. HRMS (ESI) calcd for $[C_{18}H_{30}N_2O+H]^+$ 291.2431, found 291.2435.

N-hexanoyl-11-butyl matrine derivative (**12**)

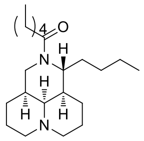

Yellow oil 1.00 g, yield 91%. ^{1}H NMR (400 MHz, $CDCl_3$) δ 4.05 (d, J = 8.0 Hz, 1H), 3.66 – 3.32 (m, 1H), 2.74 (s, 2H), 2.32 – 2.29 (m, 3H), 2.01 (s, 1H), 1.83 – 1.26 (m, 24H), 0.90 – 0.88 (d, J = 6.1 Hz, 6H). ^{13}C NMR (100 MHz, $CDCl_3$) δ 177.7, 62.7, 56.3, 53.9, 39.8, 34.3, 33.2, 31.7, 31.3, 29.0, 28.6, 24.9, 24.6,

22.8, 22.5, 22.3, 21.2, 20.9, 14.1, 14.0, 13.9. HRMS (ESI) calcd for $[C_{21}H_{38}N_2O+H]^+$ 335.3057, found 335.3061.

N-cyclopropylformyl-11- butyl matrine derivative (**13**)

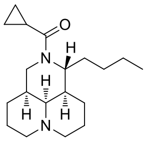

Yellow oil 0.38 g, yield 90%.^{1}H NMR (400 MHz, $CDCl_3$) δ 4.06 – 4.00 (m, 1H), 3.95 (s, 1H), 3.33 (s, 1H), 2.75 (s, 2H), 2.05 (s, 1H), 1.88 – 1.26 (m, 18H), 1.04 – 0.65 (m, 8H).^{13}C NMR (100 MHz, $CDCl_3$) δ 172.8, 62.7, 56.46, 56.43, 54.64, 39.9, 34.5, 33.5, 29.6, 28.8, 28.6, 22.8, 21.2, 20.8, 14.0, 12.3, 8.2, 6.9, 6.4. HRMS (ESI) calcd for $[C_{19}H_{32}N_2O+H]^+$ 305.2587, found 305.2591.

N-acetyl-11-butyl matrine derivative (**14**)

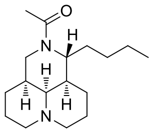

Yellow oil 0.75 g, yield 90%. ^{1}H NMR (400 MHz, $CDCl_3$) δ 4.15 (s, 1H), 3.44 (s, 1H), 2.64 (d, *J* = 8.0 Hz, 2H), 2.02 (s, 3H), 1.98 – 1.26 (m, 20H), 0.84 (d, *J* = 8.0 Hz, 3H). ^{13}C NMR (100 MHz, $CDCl_3$) δ 169.9, 62.4, 62.0, 56.0, 55.5, 52.9, 45.3, 40.9, 39.9, 38.1, 34.3, 33.4, 29.2, 28.6, 22.8, 21.0, 14.0. HRMS (ESI) calcd for $[C_{17}H_{30}N_2O+H]^+$ 279.2431, found 279.2435.

N-cyclohexylformyl-11-butyl matrine derivative (**15**)

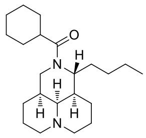

Light yellow solid 1.40 g, yield 97%. Mp 102–103 °C. ^{1}H NMR (400 MHz, $CDCl_3$) δ 4.10 (d, *J* = 8.0 Hz, 2H), 3.63 (d, *J* = 16.0 Hz, 1H), 3.49 (d, *J* = 12.0 Hz, 2H), 3.24 (s, 1H), 2.67 (s, 2H), 2.41 – 2.31 (m, 4H), 2.10 (d, *J* = 12.0 Hz, 2H), 1.94 (s, 1H), 1.80 – 1.20 (m, 20H), 0.86 (d, *J* = 4.0 Hz, 3H). ^{13}C NMR (100 MHz, $CDCl_3$) δ 178.7, 65.6, 56.7, 56.3, 56.1, 48.7, 42.4, 38.0, 37.5, 30.6, 29.8, 28.7, 28.4, 26.0, 25.8, 25.6, 25.1, 22.7, 19.0, 18.7, 14.1. HRMS (ESI) calcd for $[C_{22}H_{38}N_2O+H]^+$ 347.3057, found 347.3058.

The single crystal diffraction analysis of the hydrochloride of compound **15** showed that after D-ring was opened, the optical configuration of chiral centers in 5-C, 6-C, 7-C and 11-C were all unchanged, and the A, B and C ring of the compound still existed in the chair form. (CCDC 1531045)

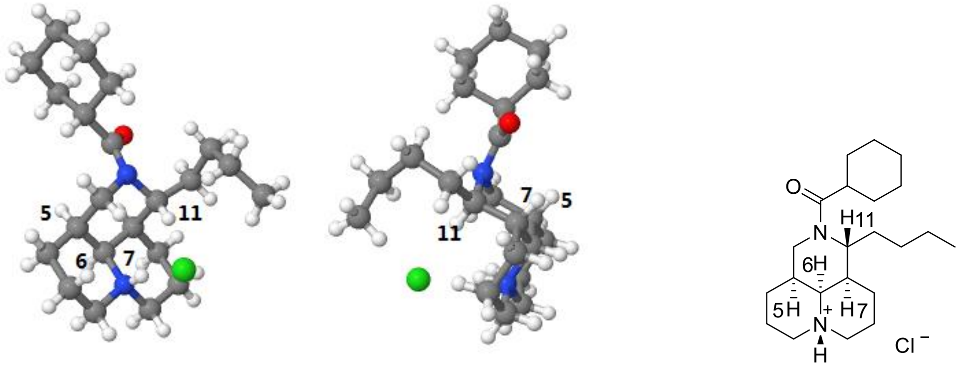

a. Front view b. Side view **IV-15** hydrochloride

N-benzyl-11-butyl matrine derivative (**16**)

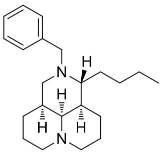

White oil 0.30 g，yield 79%. ^{1}H NMR (400 MHz, $CDCl_3$) δ 7.34 – 7.20 (m, 5H), 4.09 (d, J = 12.0 Hz, 1H), 3.04 (d, J = 12.0 Hz, 1H), 2.83 – 2.75 (m, 2H), 2.55 (t, J = 12.0 Hz, 1H), 2.32 – 2.28 (m, 1H), 2.01 (s, 1H), 1.96 – 1.23 (m, 19H), 0.87 (t, J = 4.0 Hz, 3H). ^{13}C NMR (100 MHz, $CDCl_3$) δ 128.8, 128.5, 128.1, 126.9, 126.5, 64.6, 57.6, 57.4, 57.0, 52.2, 38.5, 34.2, 29.7, 29.2, 28.2, 27.4, 26.2, 23.2, 21.6, 21.4, 14.1. HRMS (ESI) calcd for $[C_{22}H_{34}N_2+H]^+$ 327.2795, found 327.2798.

N-(1-naphthylmethyl)-11-butyl matrine derivative (**17**)

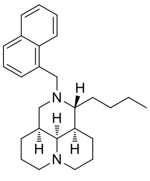

Light yellow oil 0.26 g, yield 62%. ^{1}H NMR (400 MHz, $CDCl_3$) δ 8.41 (d, J = 8.0 Hz, 1H), 7.86 – 7.72 (m, 2H), 7.56 – 7.39 (m, 4H), 4.56 (d, J = 12.0 Hz, 1H), 3.52 (d, J = 12.0 Hz, 1H), 2.86 (d, J = 8.0 Hz, 2H), 2.77 (d, J = 8.0 Hz, 1H), 2.62 (t, J = 12.0 Hz, 1H), 2.24 (d, J = 12.0 Hz, 1H), 2.03 – 1.23

(m, 19H), 0.90 (t, *J* = 4.0 Hz, 3H). ^{13}C NMR (100 MHz, $CDCl_3$) *δ* 136.1, 133.7, 132.5, 128.4, 127.1, 126.8, 125.5, 125.4, 124.5, 64.7, 59.1, 57.7, 57.4, 55.8, 52.6, 38.8, 34.6, 29.3, 28.2, 27.3, 26.8, 23.3, 21.7, 21.6, 14.2. HRMS (ESI) calcd for $[C_{26}H_{36}N_2+H]^+$ 377.2951, found 377.2944.

N-n-hexyl -11-butyl matrine derivative (**18**)

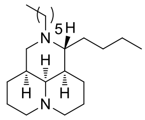

Light yellow oil 0.40 g, yield 78%. ^{1}H NMR (400 MHz, $CDCl_3$) *δ* 4.31 – 4.14 (m, 1H), 3.48 – 2.80 (m, 8H), 2.31 – 1.25 (m, 25H), 0.96 – 0.90 (m, 6H).^{13}C NMR (100 MHz, $CDCl_3$) *δ* 100.0, 62.4, 59.0, 55.8, 53.8, 50.2, 36.0, 31.1, 30.6, 27.9, 27.2, 26.6, 25.0, 24.6, 23.2, 22.6, 22.4, 18.4, 18.4, 13.9, 13.6. HRMS (ESI) calcd for $[C_{21}H_{40}N_2+H]^+$ 321.3264, found 321.3258.

N-cyclohexylmethyl-11-butyl matrine derivative (**19**)

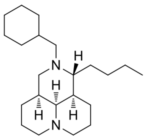

White solid 0.5 g, yield 89%. Mp 89–90 °C. ^{1}H NMR (400 MHz, $CDCl_3$) *δ* 2.80 (dd, *J* = 12.0, 8.0 Hz, 2H), 2.61 – 2.48 (m, 4H), 2.10 – 1.05 (m, 30H), 0.90 (t, *J* = 8.0 Hz, 3H), 0.92 – 0.78 (m, 1H).^{13}C NMR (100 MHz, $CDCl_3$) *δ* 64.6, 59.9, 58.1, 57.6, 57.4, 53.1, 38.7, 35.9, 34.3, 32.5 , 29.5, 28.5, 27.3, 26.8, 26.6, 26.4, 23.3, 23.3, 21.7, 21.5, 14.1. HRMS (ESI) calcd for $[C_{22}H_{40}N_2O+H]^+$ 333.3264, found 333.3268.

N-[12-(11-butyl matrine)]-*N′*-cyclohexyl urea (**20**)

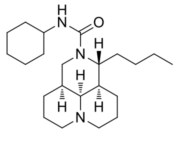

White solid 0.24 g. Mp 86–87 °C. ^{1}H NMR (400 MHz, $CDCl_3$) *δ* 4.35 (d, *J* = 8.0 Hz, 1H), 3.72 – 3.60 (m, 2H), 3.52 (dd, *J* = 12.0, 4.0 Hz, 1H), 3.19 (dd, *J* = 8.0, 4.0 Hz, 1H), 2.74 (d, *J* = 4.0 Hz, 2H), 2.04 (s, 1H), 1.95 – 1.07 (m, 28H), 0.89 (t, *J* = 4.0 Hz, 3H). ^{13}C NMR (100 MHz, $CDCl_3$) *δ*

158.3, 63.6, 56.8, 55.3, 48.9, 46.8, 39.1, 34.8, 34.1, 33.9, 32.2, 28.7, 28.5, 28.1, 25.7, 25.0, 25.0, 22.8, 21.2, 21.2, 14.1.HRMS (ESI) calcd for $[C_{22}H_{39}N_3O+H]^+$ 362.3166, found 362.3164.

N-[12-(11-deoxymatrine)]-*N′*-(3-methyl phenyl) urea (**21**)

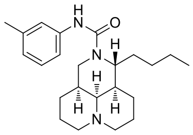

Light yellow solid 0.40 g, yield 68%. Mp 93–94 °C. ^{1}H NMR (400 MHz, $CDCl_3$) δ 7.23 (s, 1H), 7.16 – 7.05 (m, 2H), 6.81 (d, *J* = 4.0 Hz, 1H), 6.48 (s, 1H, NH), 3.82 – 3.63 (m, 2H), 3.31 (dd, *J* = 8.0, 4.0 Hz, 1H), 2.79 (s, 2H), 2.31 (s, 3H), 2.14 (s, 1H), 1.92 – 1.26 (m, 18H), 0.88 (t, *J* = 4.0 Hz, 3H). ^{13}C NMR (100 MHz, $CDCl_3$) δ 156.4, 139.5, 138.7, 128.6, 123.3, 120.0, 116.4, 63.6, 56.86, 56.82， 55.6, 47.5, 38.9, 35.3, 31.8, 28.7, 28.3, 28.0, 22.8, 21.5, 21.2, 21.1, 14.1. HRMS (ESI) calcd for $[C_{23}H_{35}N_3O+H]^+$ 370.2853, found 370.2861.

2. ^{1}H NMR and ^{13}C NMR spectra for compounds B-F， 1-21

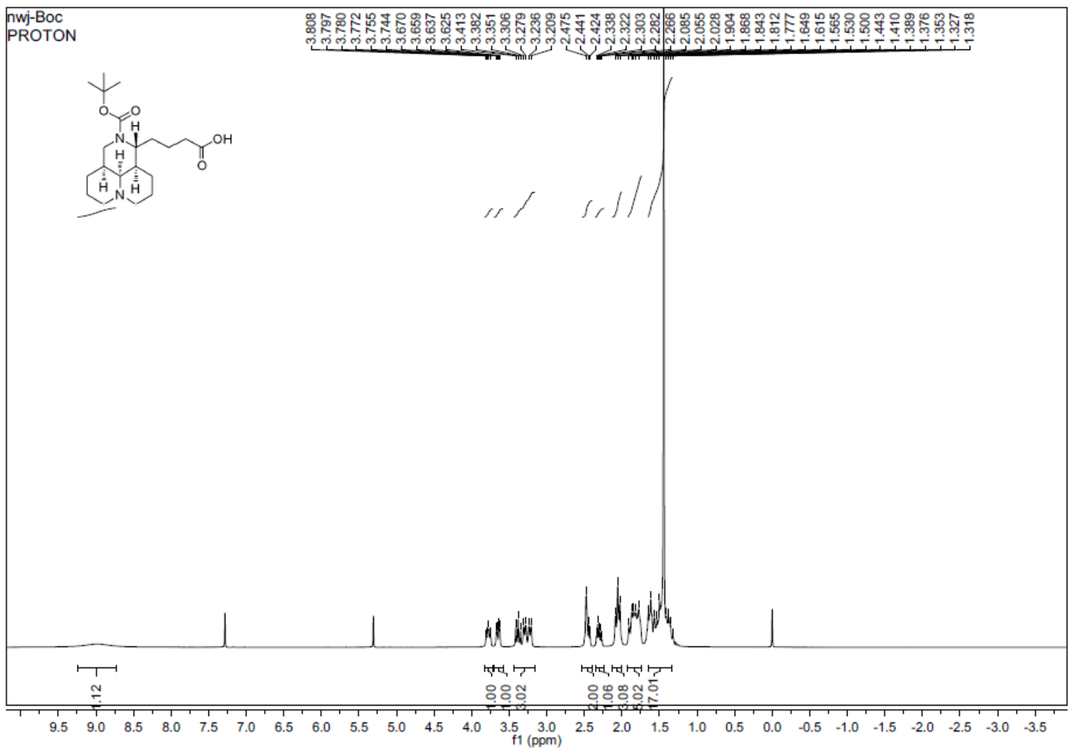

^{1}H NMR of compound B

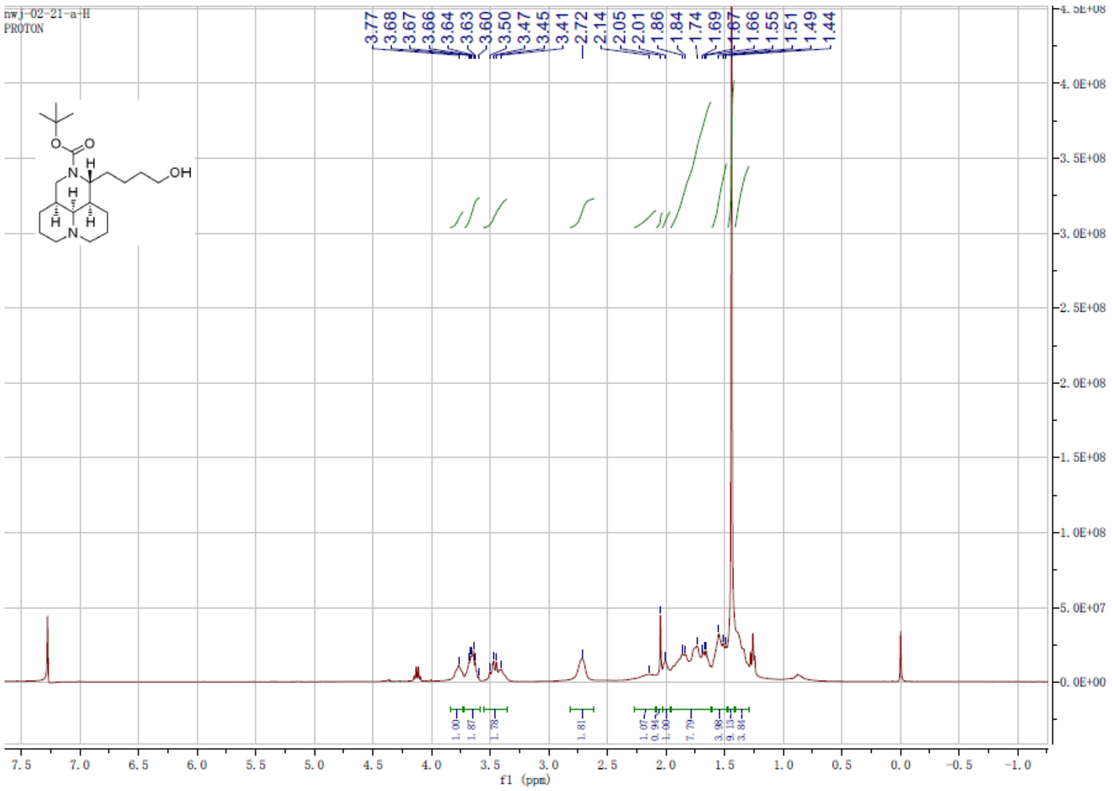

^{1}H NMR of compound C

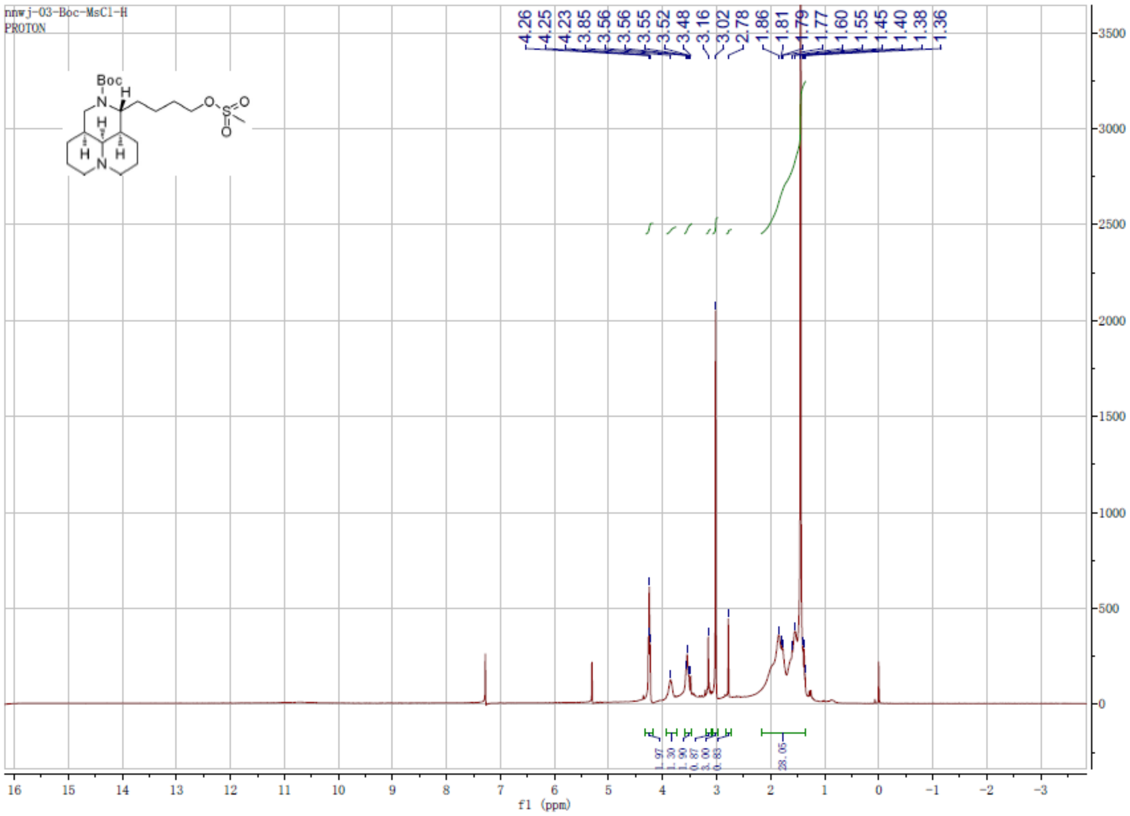

^{1}H NMR of compound D

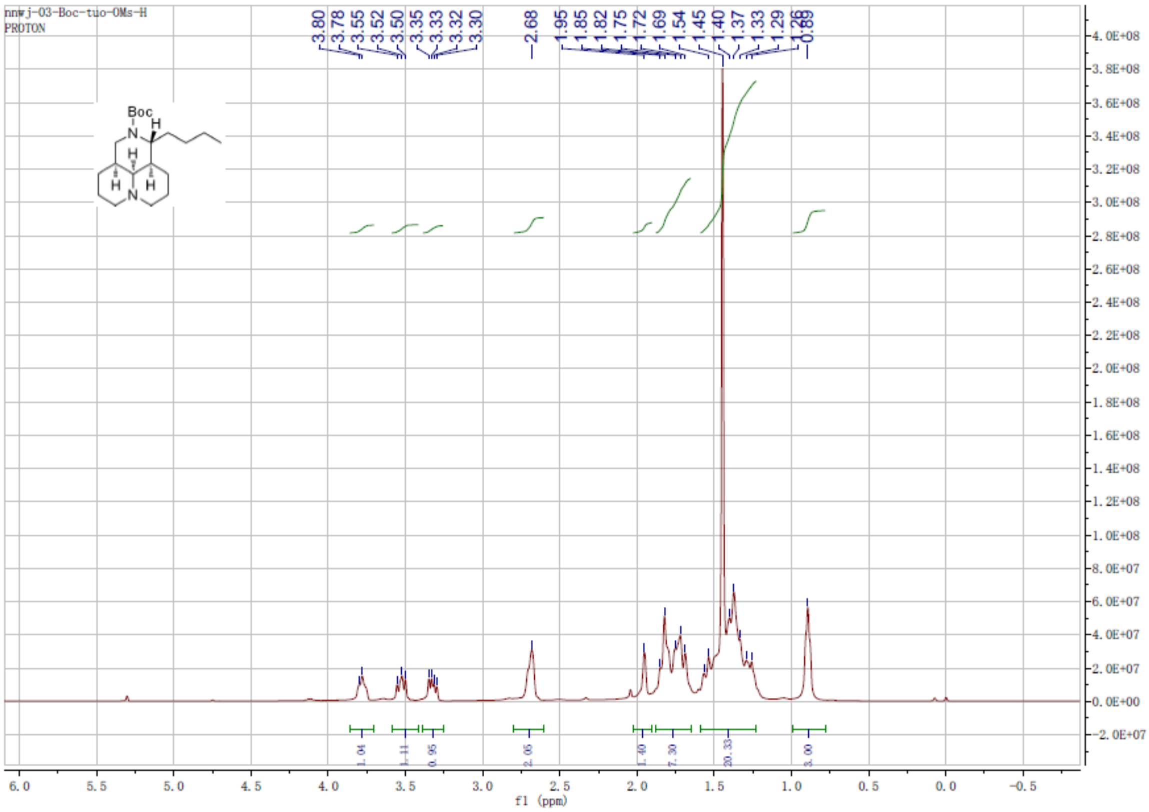

^{1}H NMR of compound E

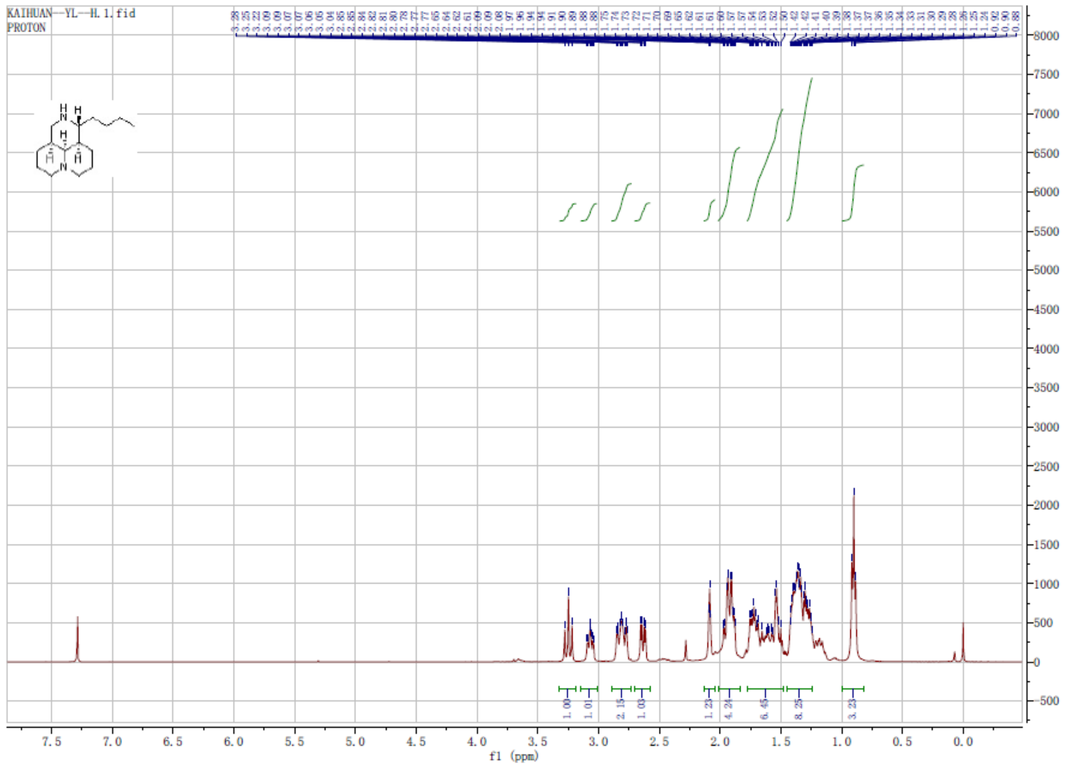

^{1}H NMR of compound F

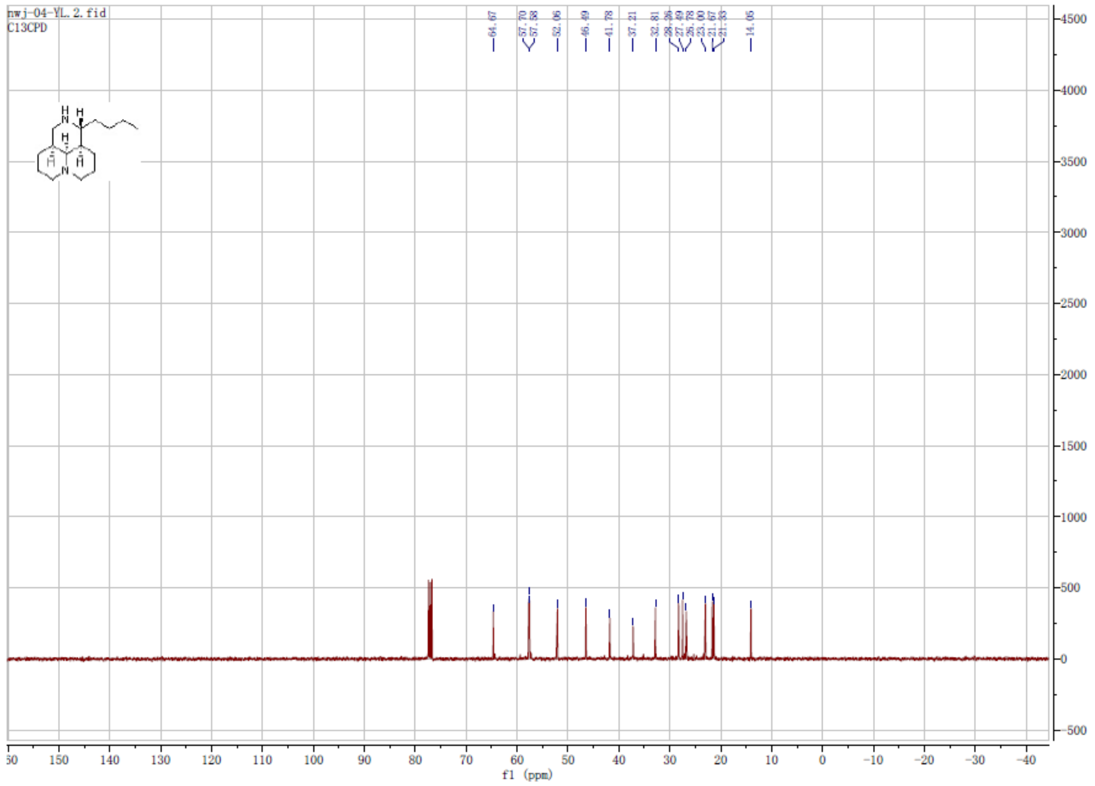

^{13}C NMR of compound F

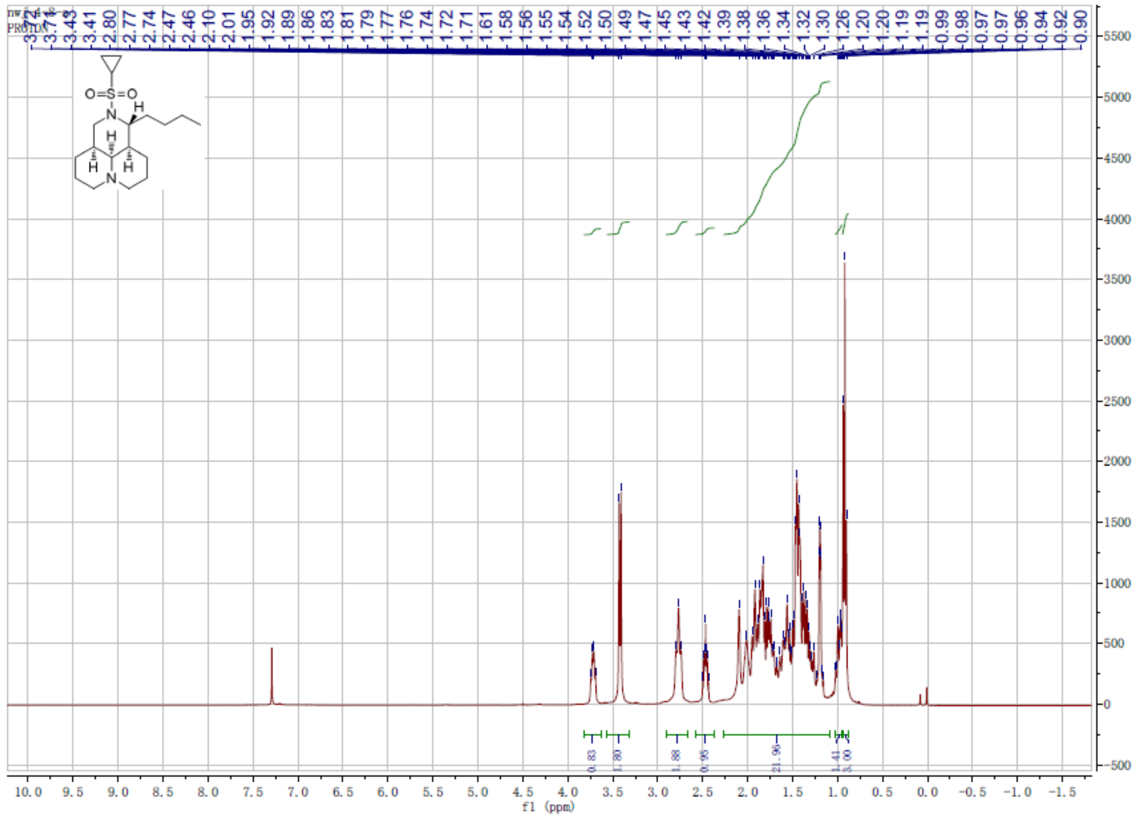

^{1}H NMR of compound 1

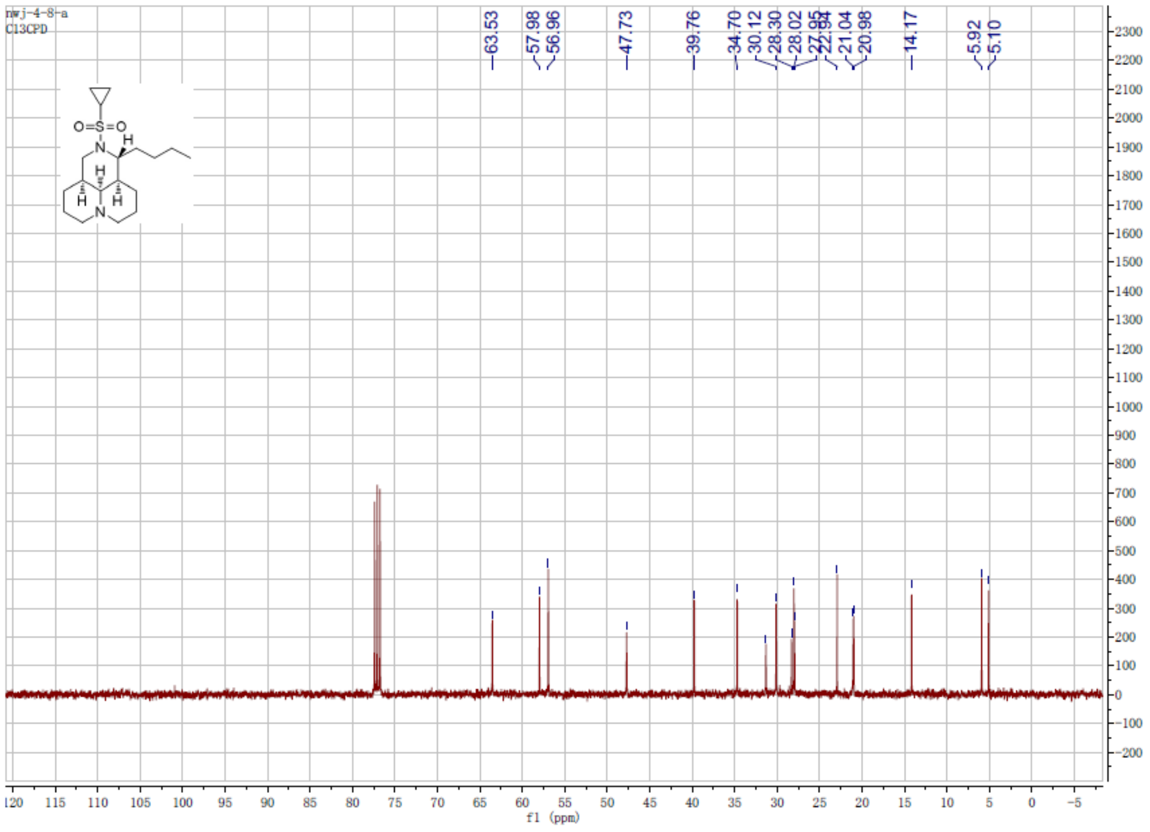

^{13}C NMR of compound 1

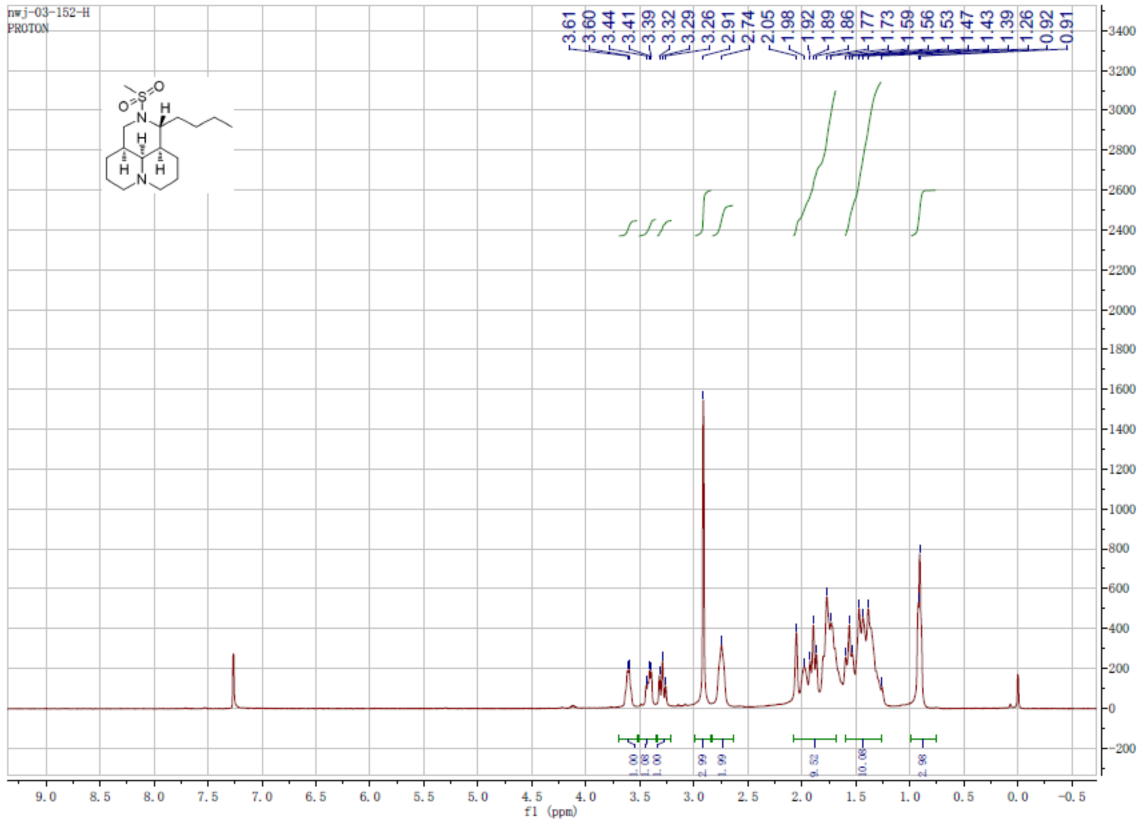

^{1}H NMR of compound 2

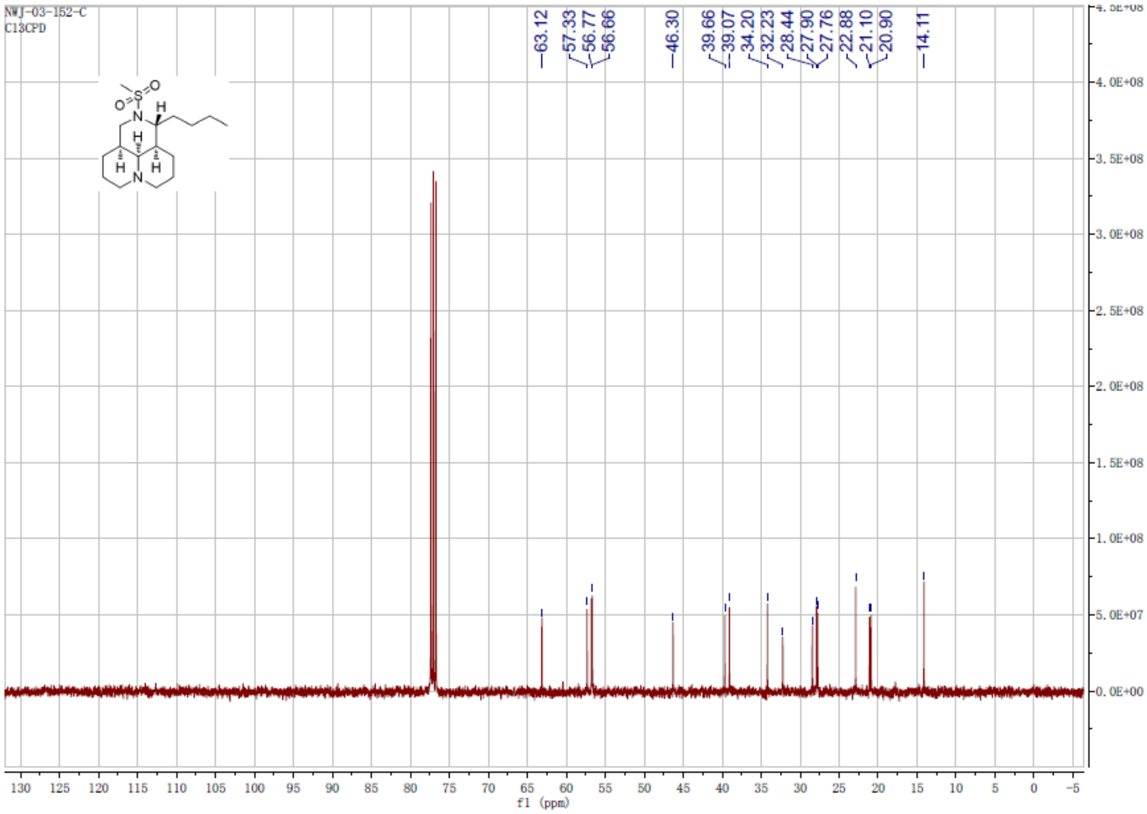

^{13}C NMR of compound 2

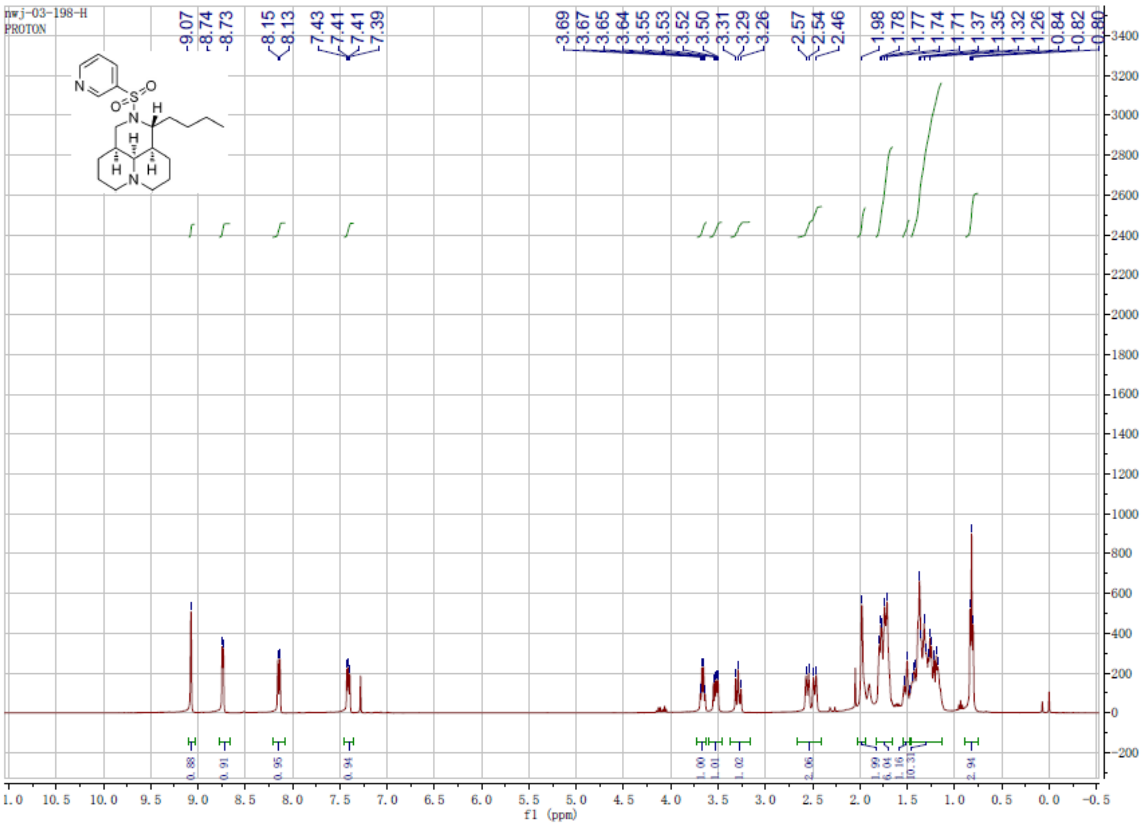

^{1}H NMR of compound 3

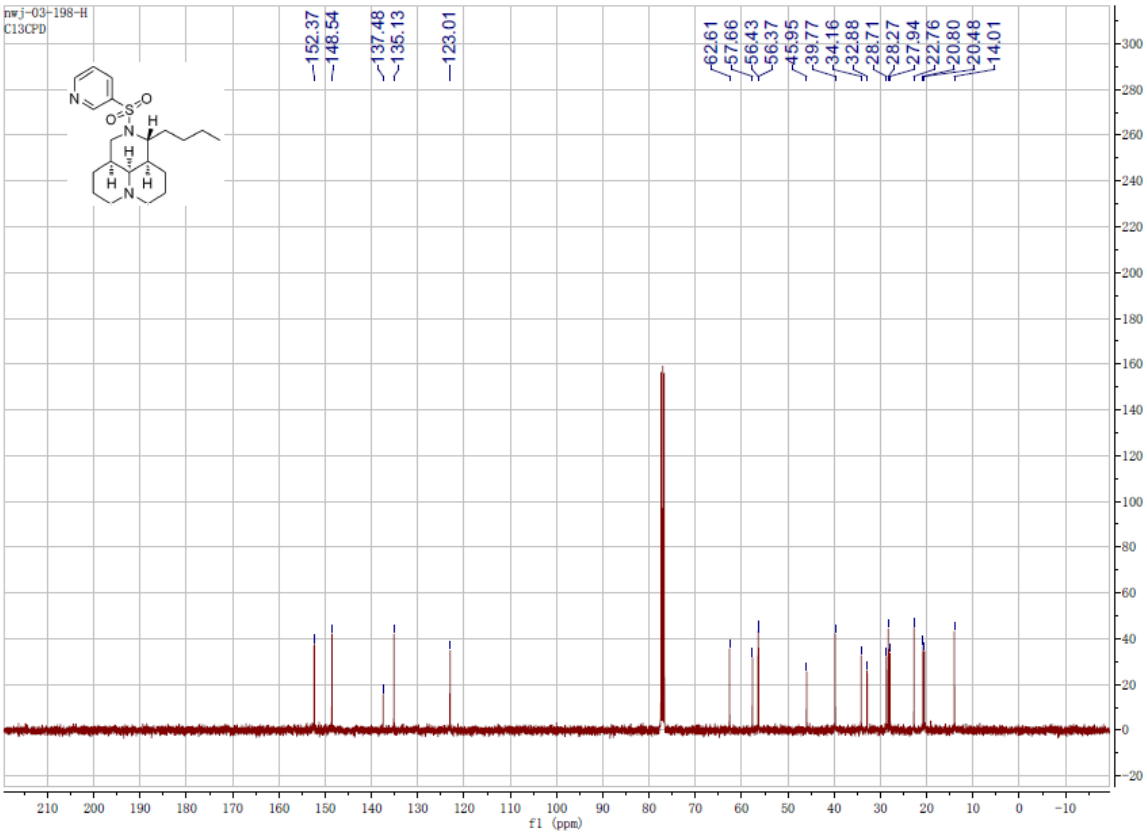

^{13}C NMR of compound 3

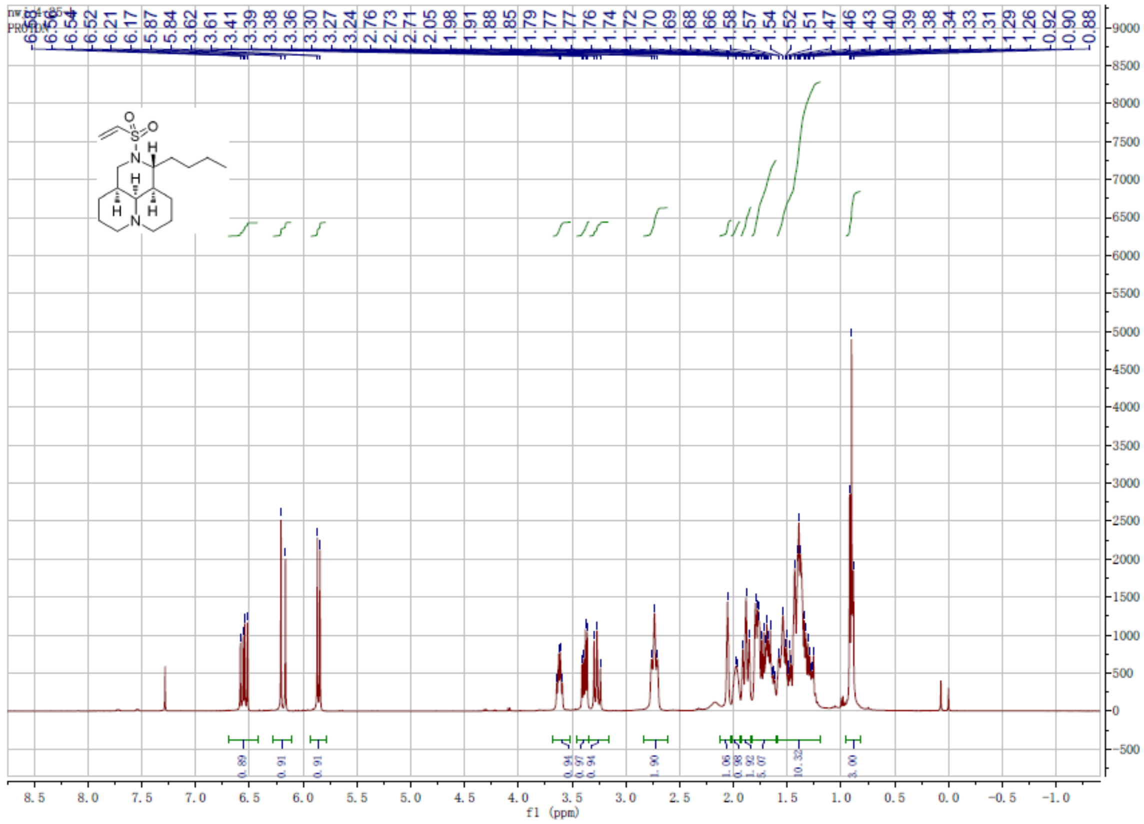

^{1}H NMR of compound 4

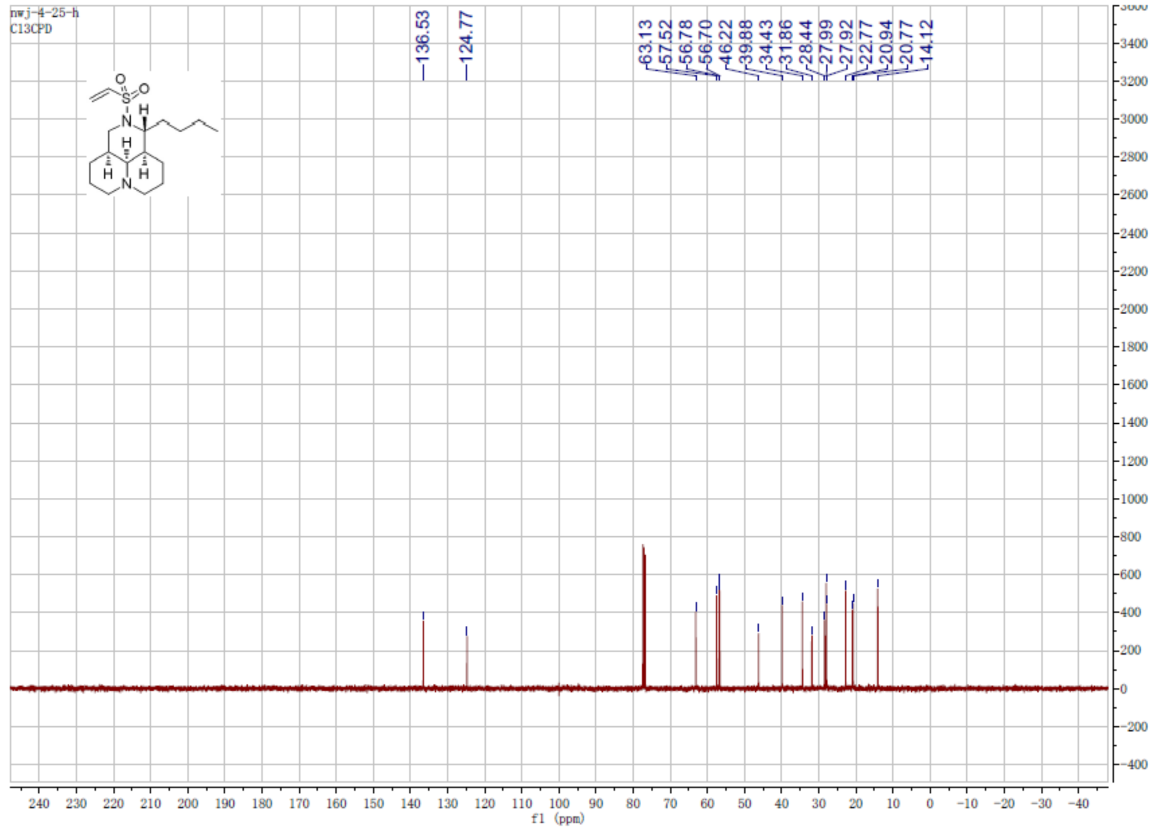

^{13}C NMR of compound 4

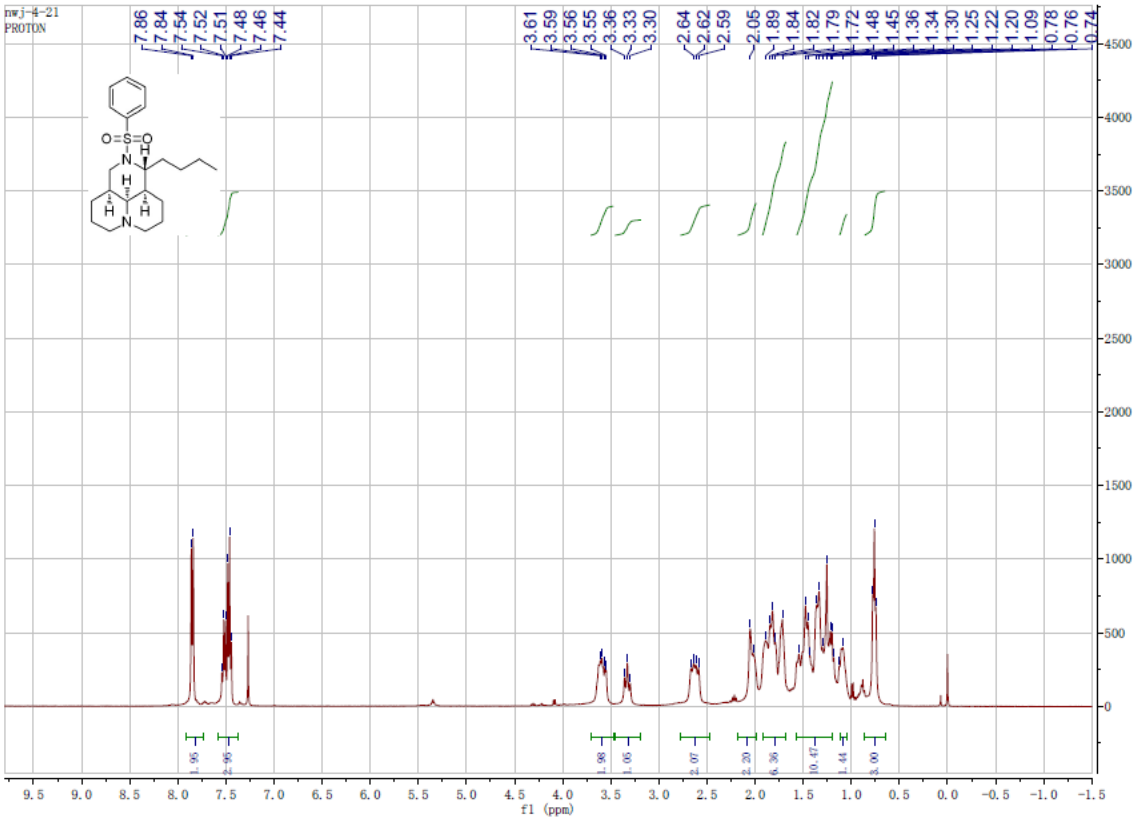

^{1}H NMR of compound 5

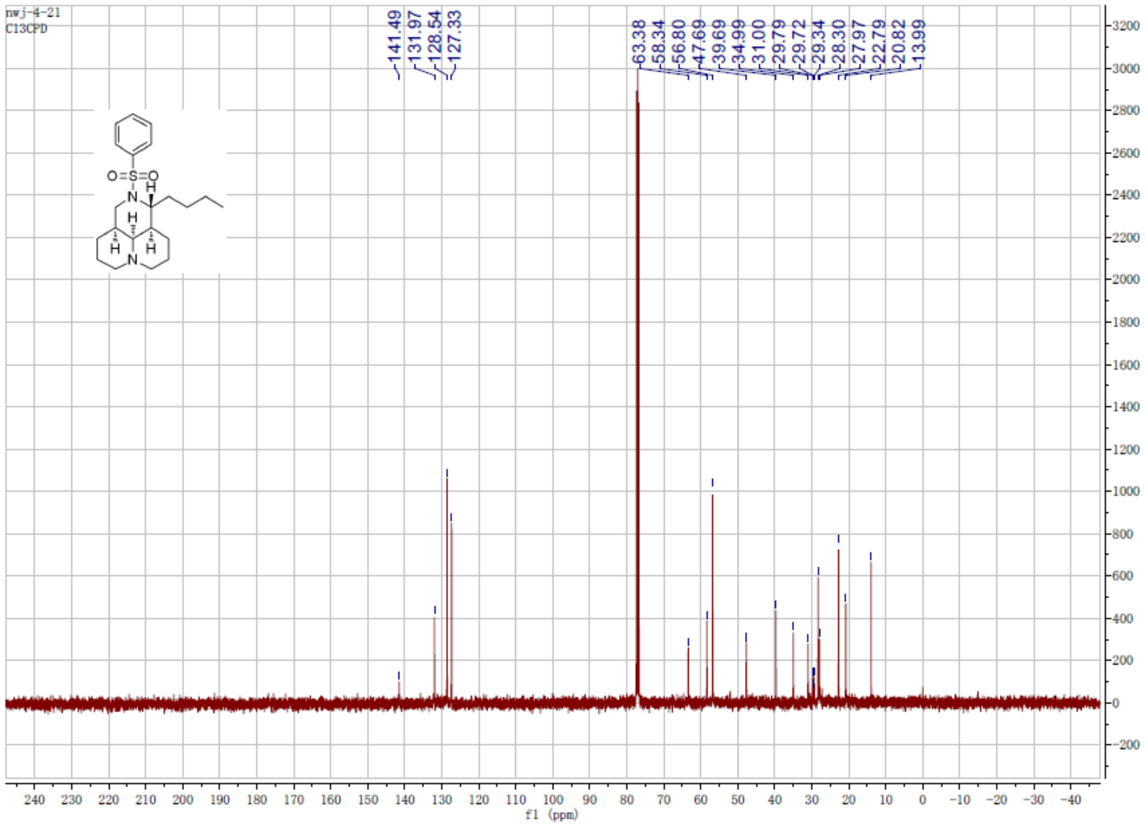

^{13}C NMR of compound 5

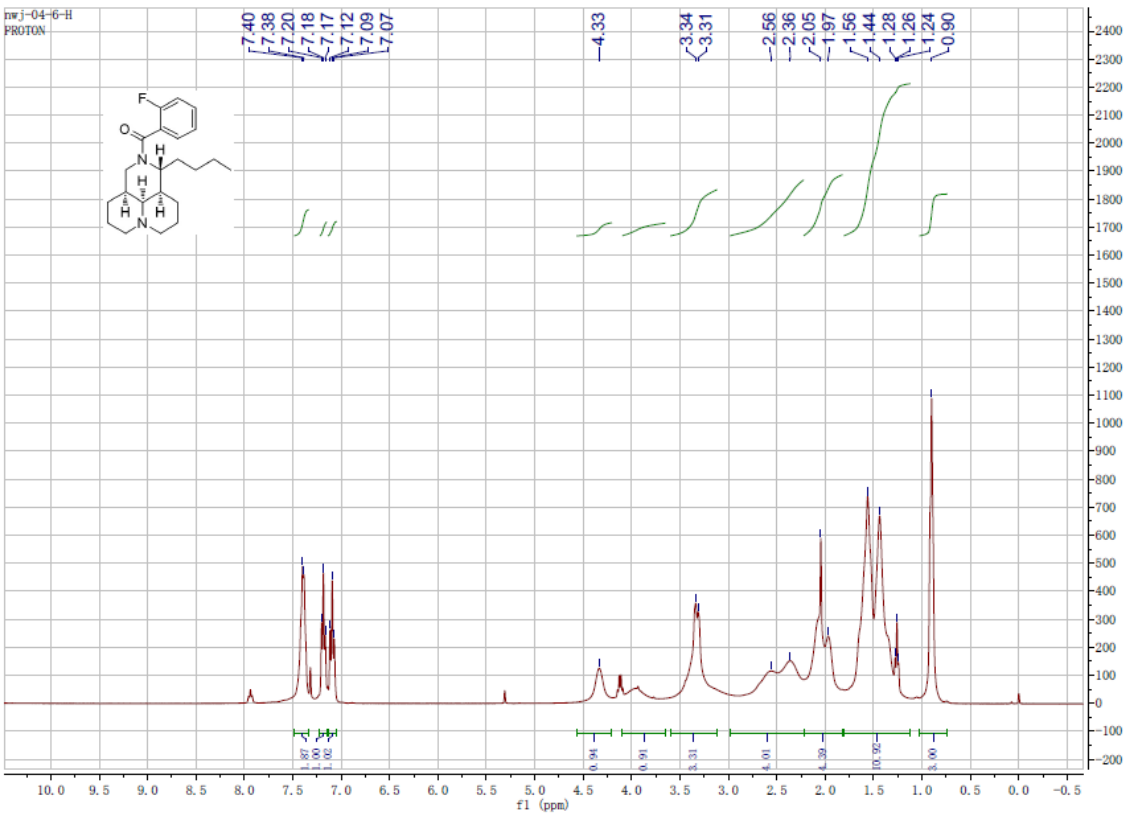

^{1}H NMR of compound 6

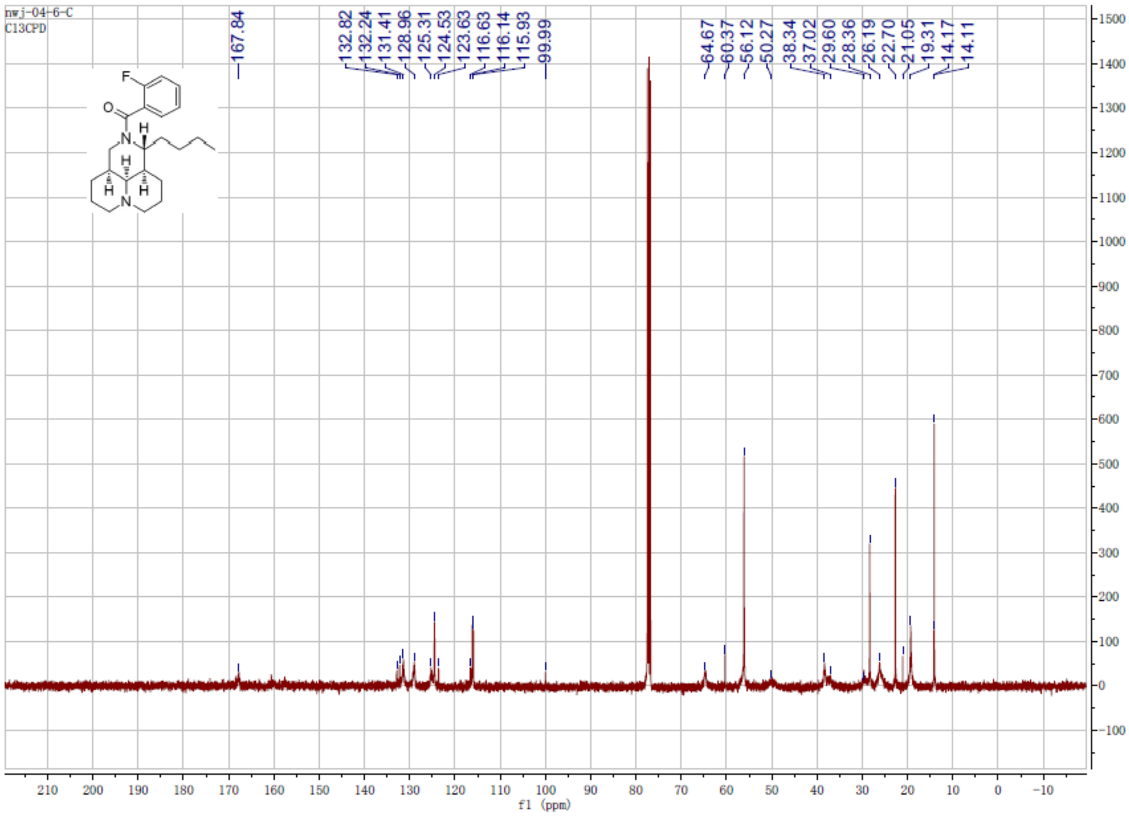

^{13}C NMR of compound 6

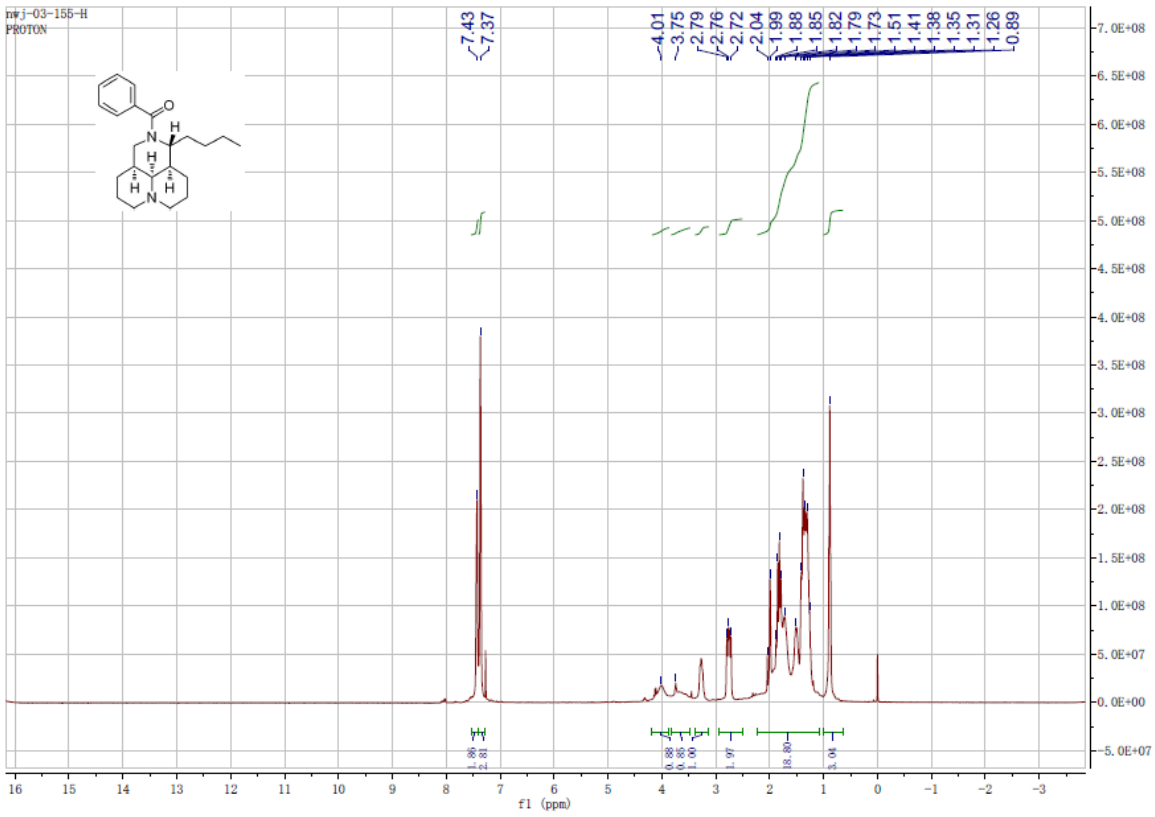

^{1}H NMR of compound 7

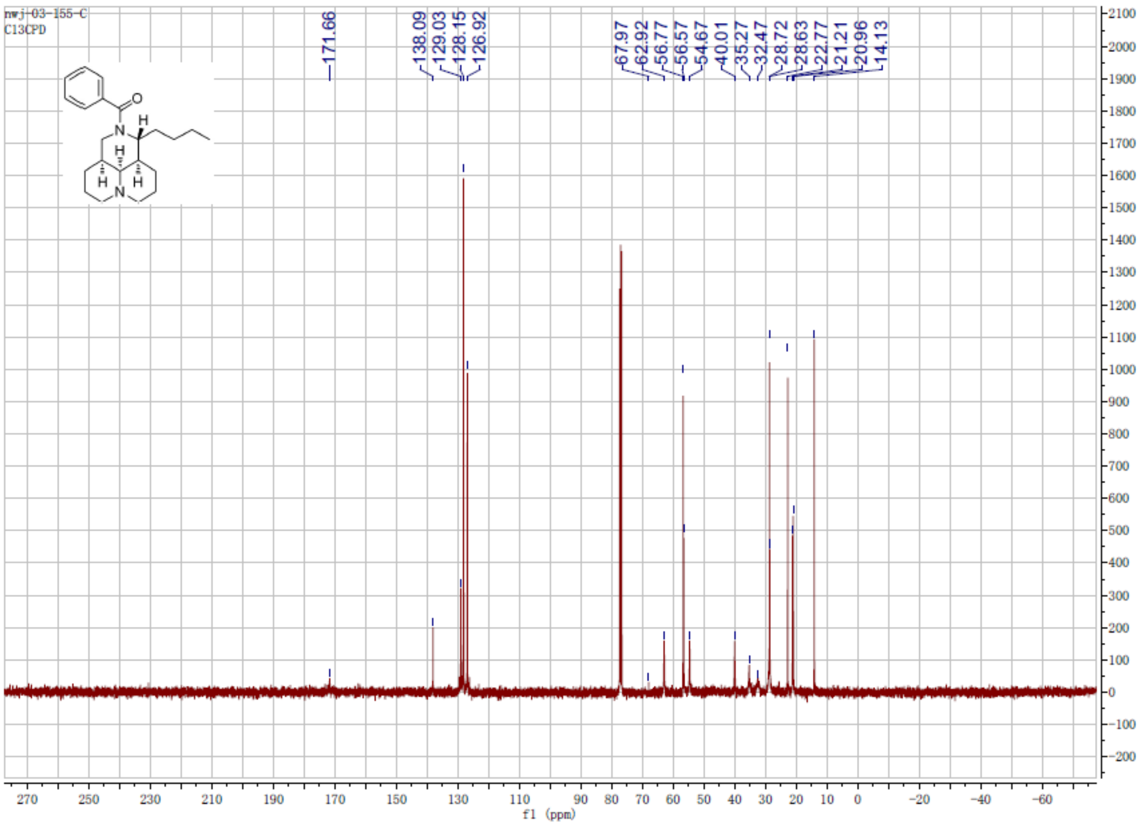

^{13}C NMR of compound 7

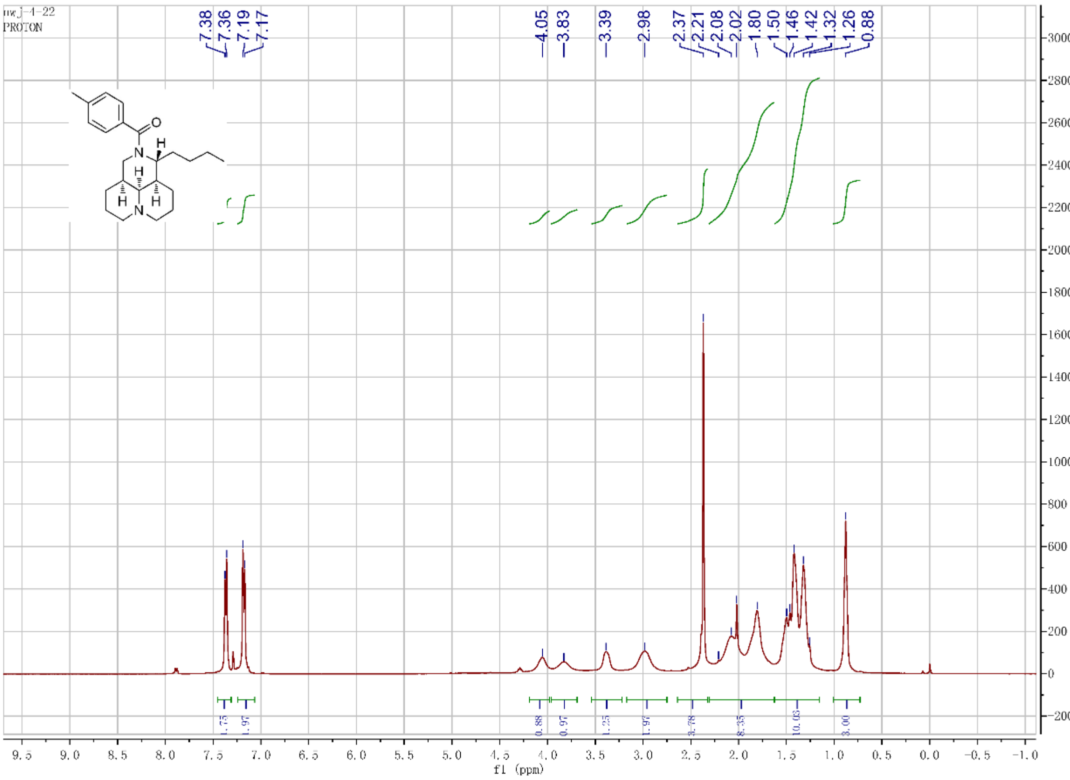

^{1}H NMR of compound 8

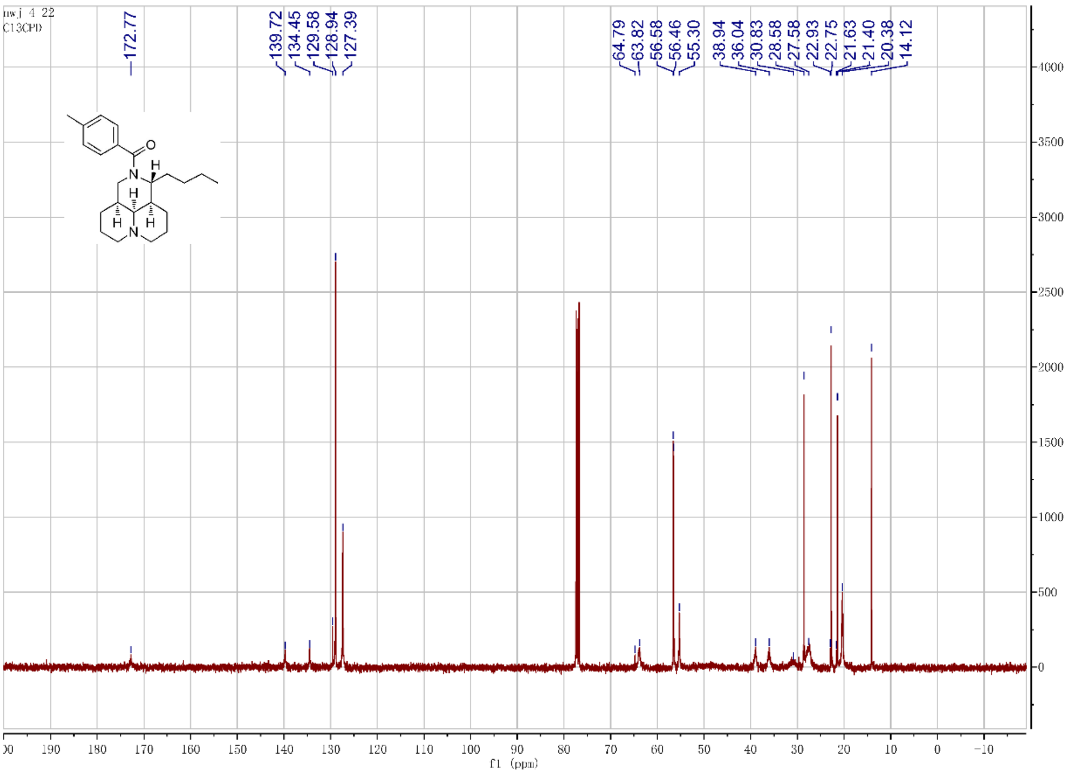

^{13}C NMR of compound 8

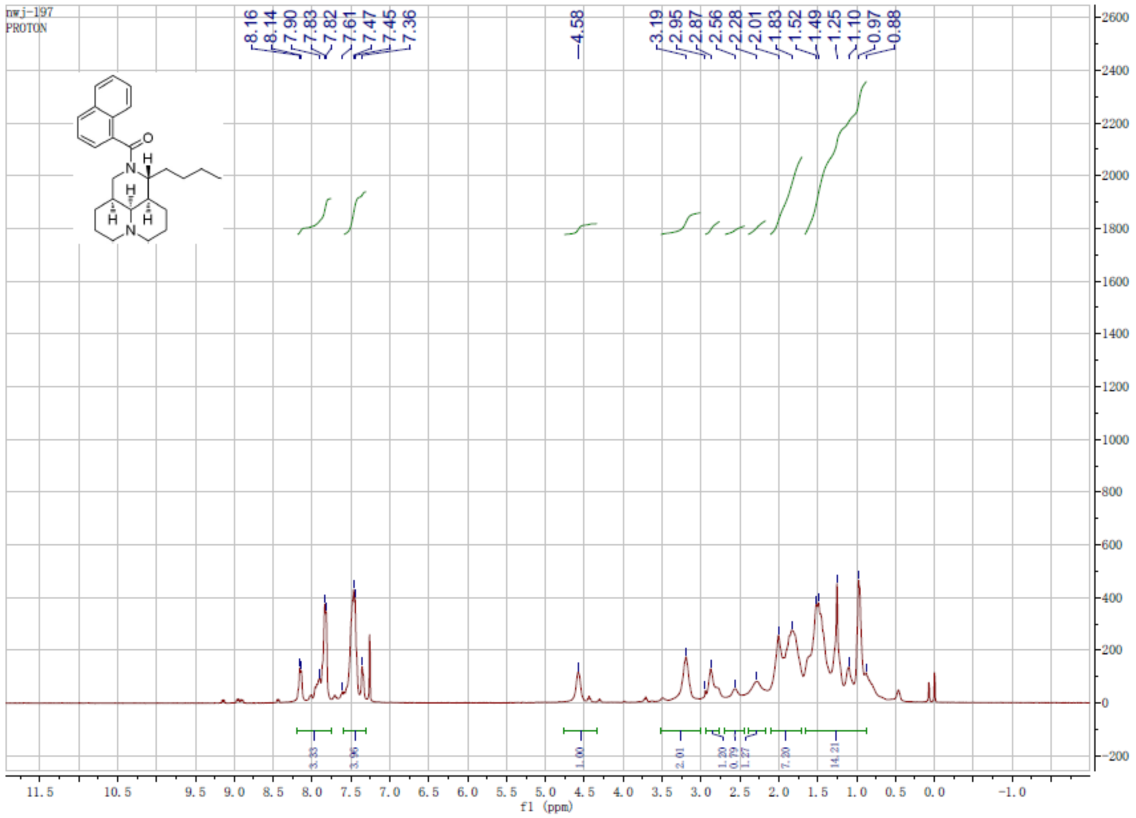

^{1}H NMR of compound 9

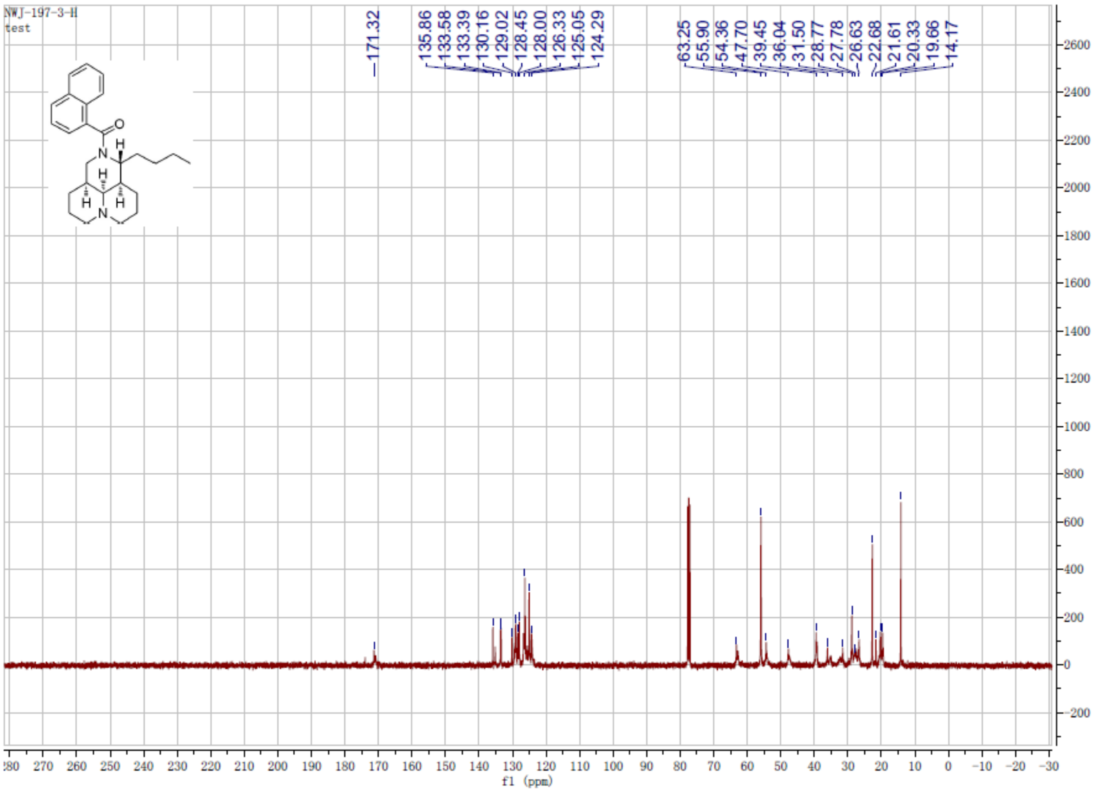

^{13}C NMR of compound 9

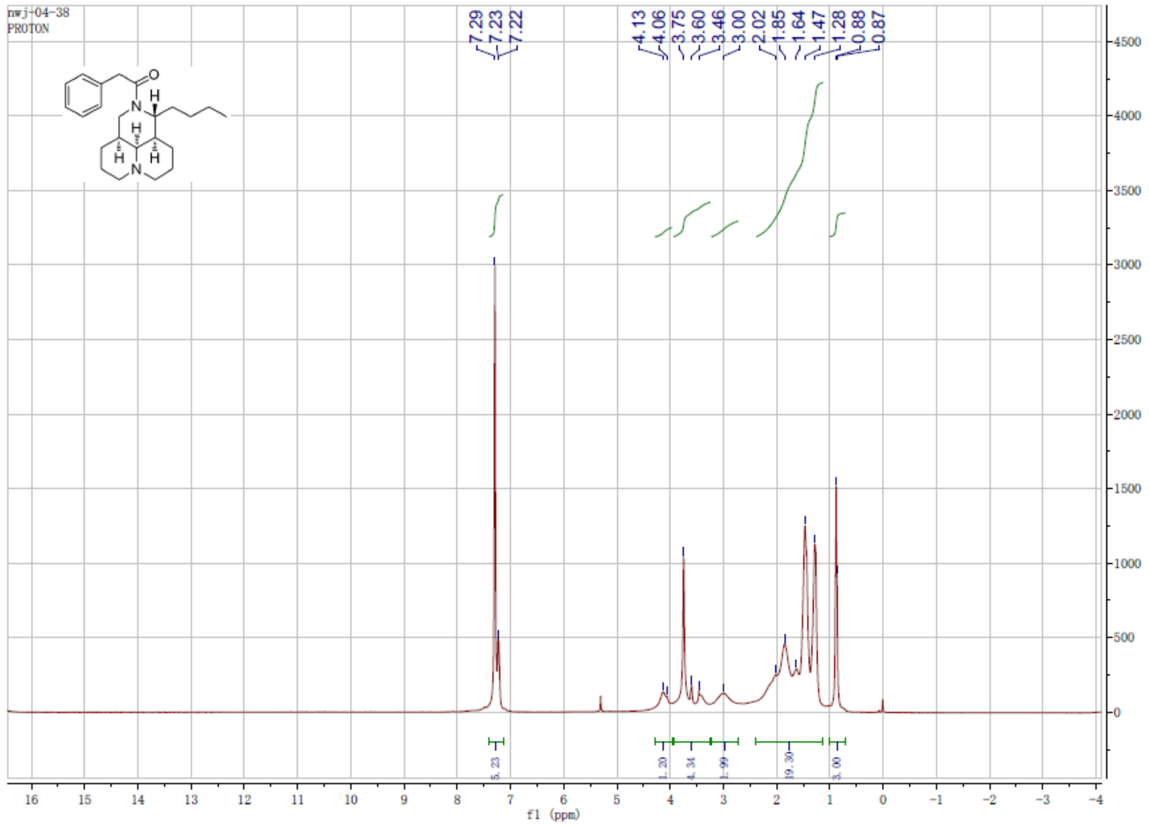

^{1}H NMR of compound 10

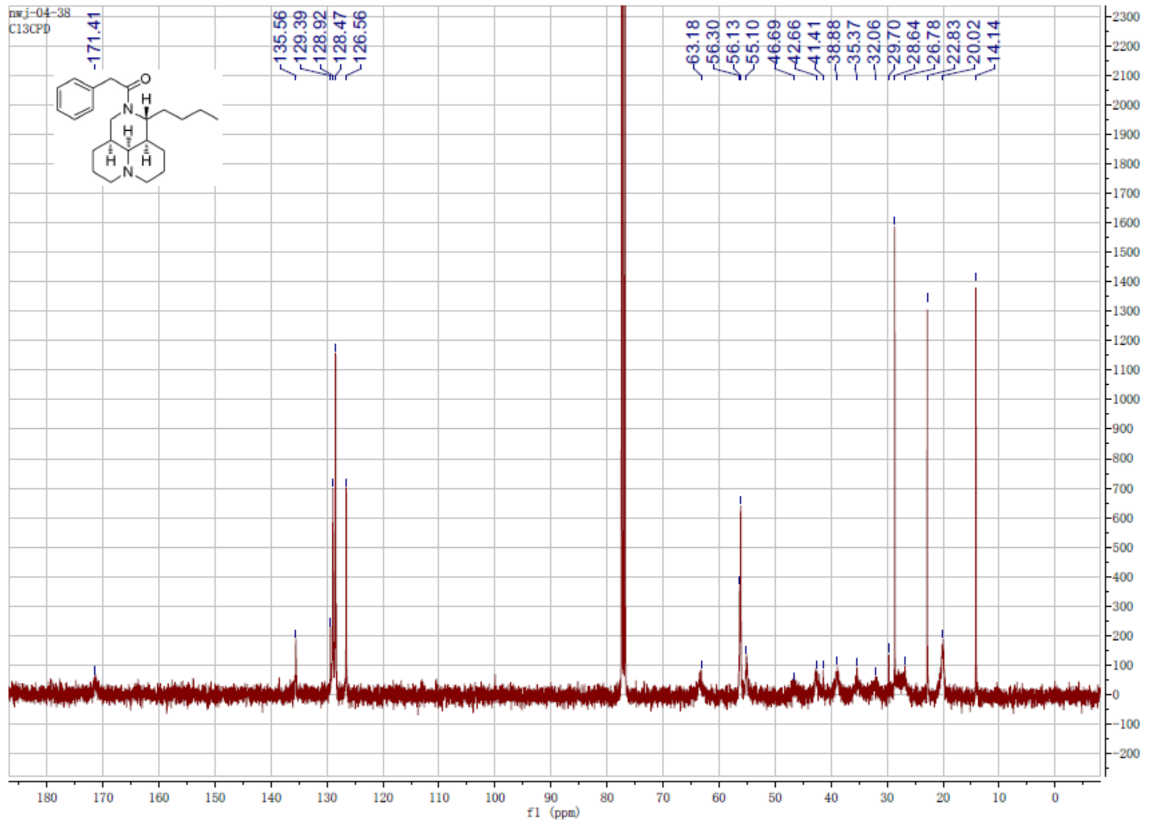

^{13}C NMR of compound 10

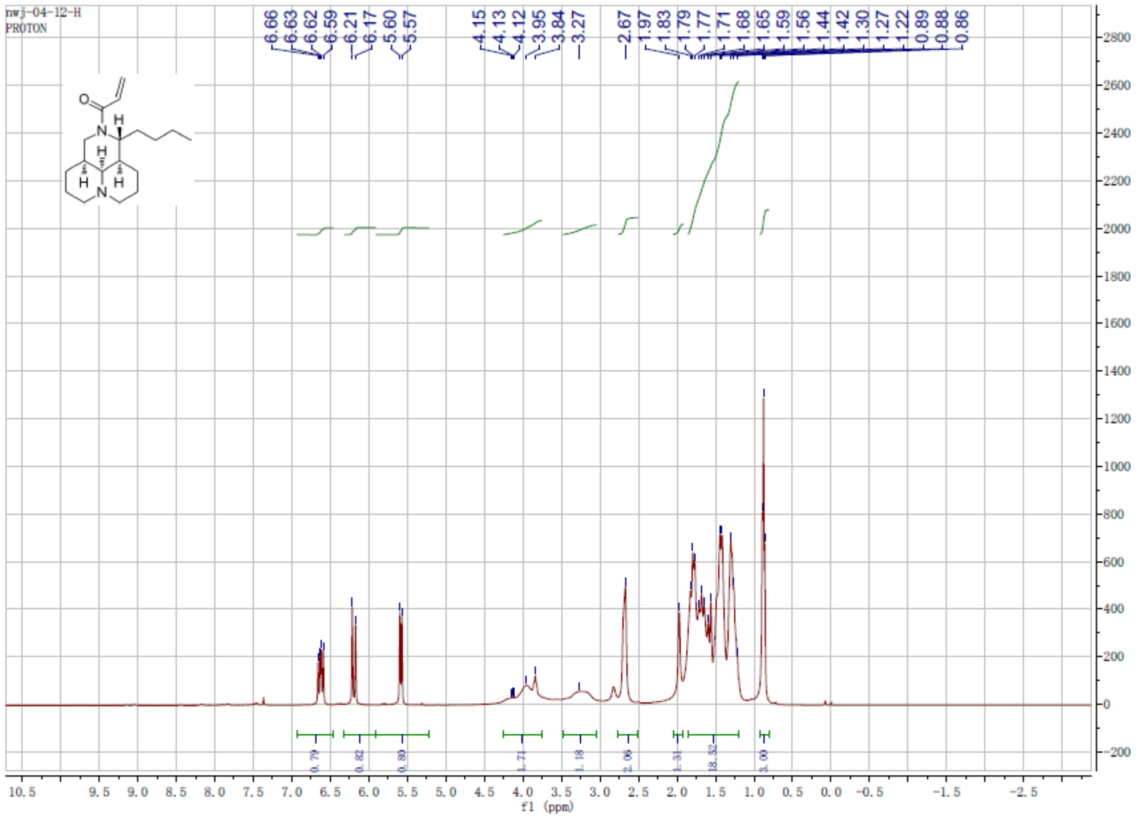

^{1}H NMR of compound 11

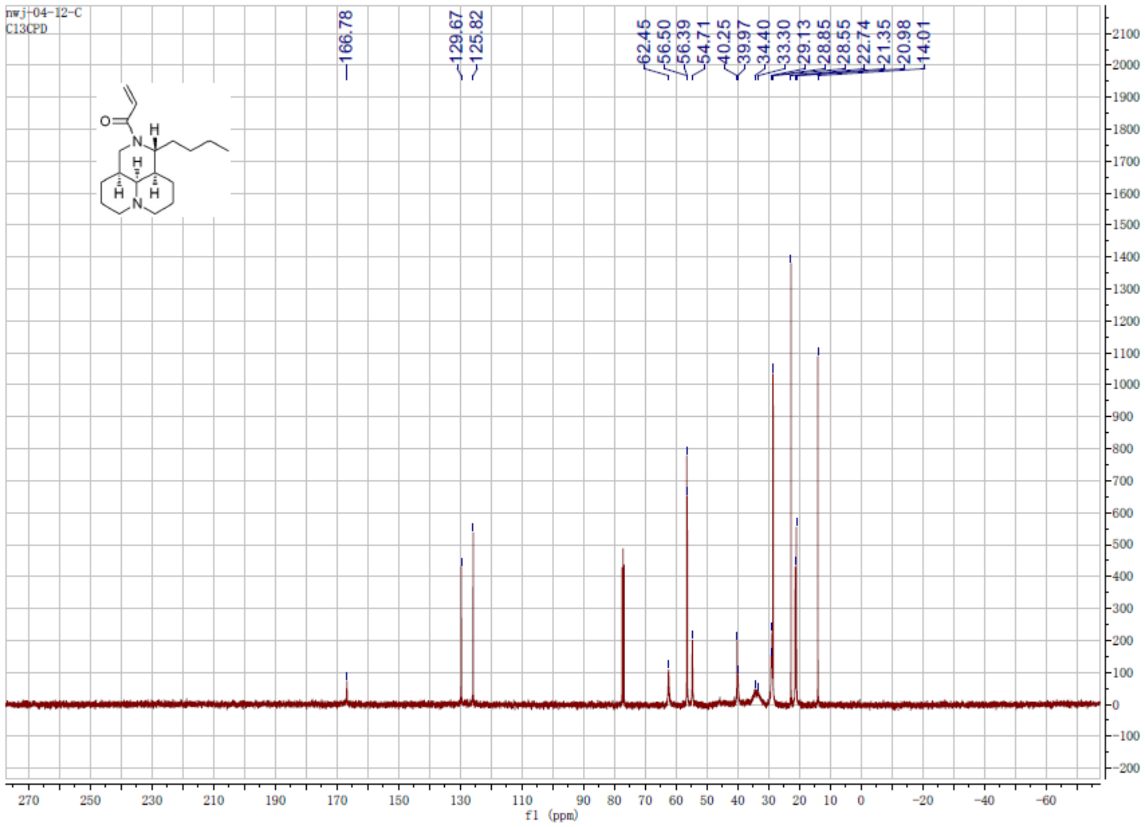

^{13}C NMR of compound 11

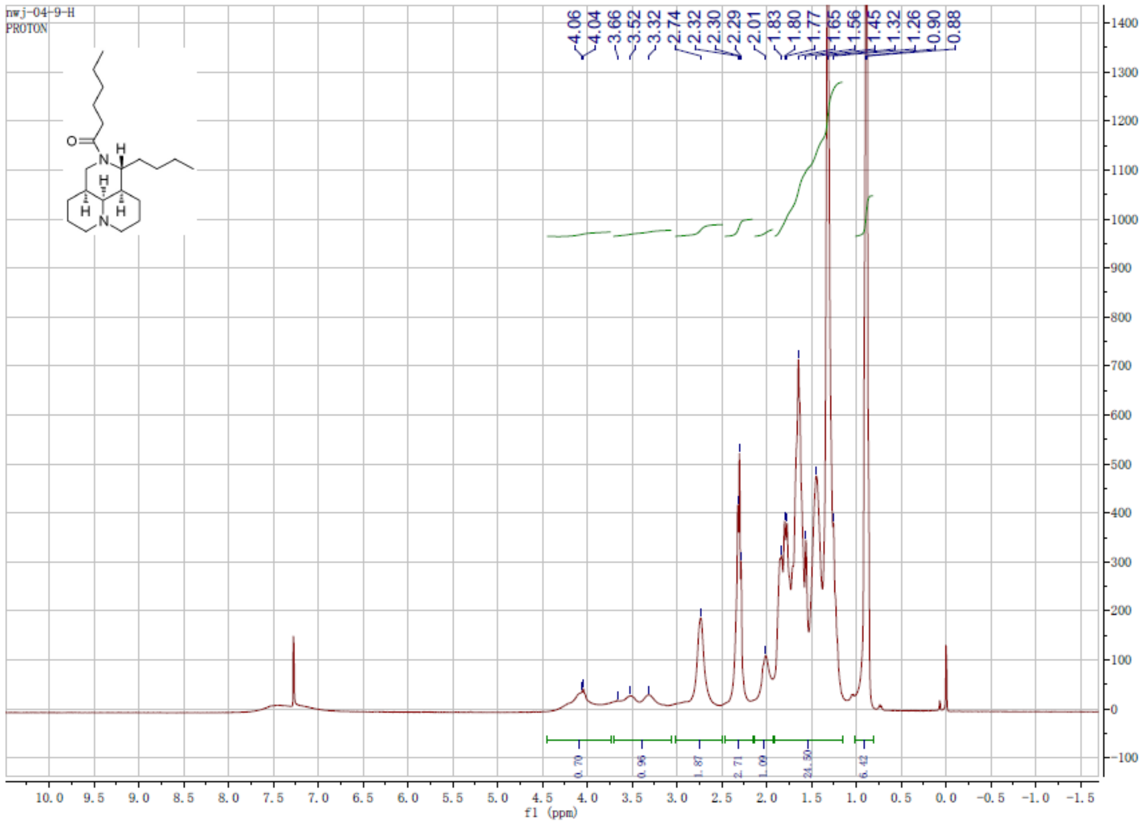

^{1}H NMR of compound 12

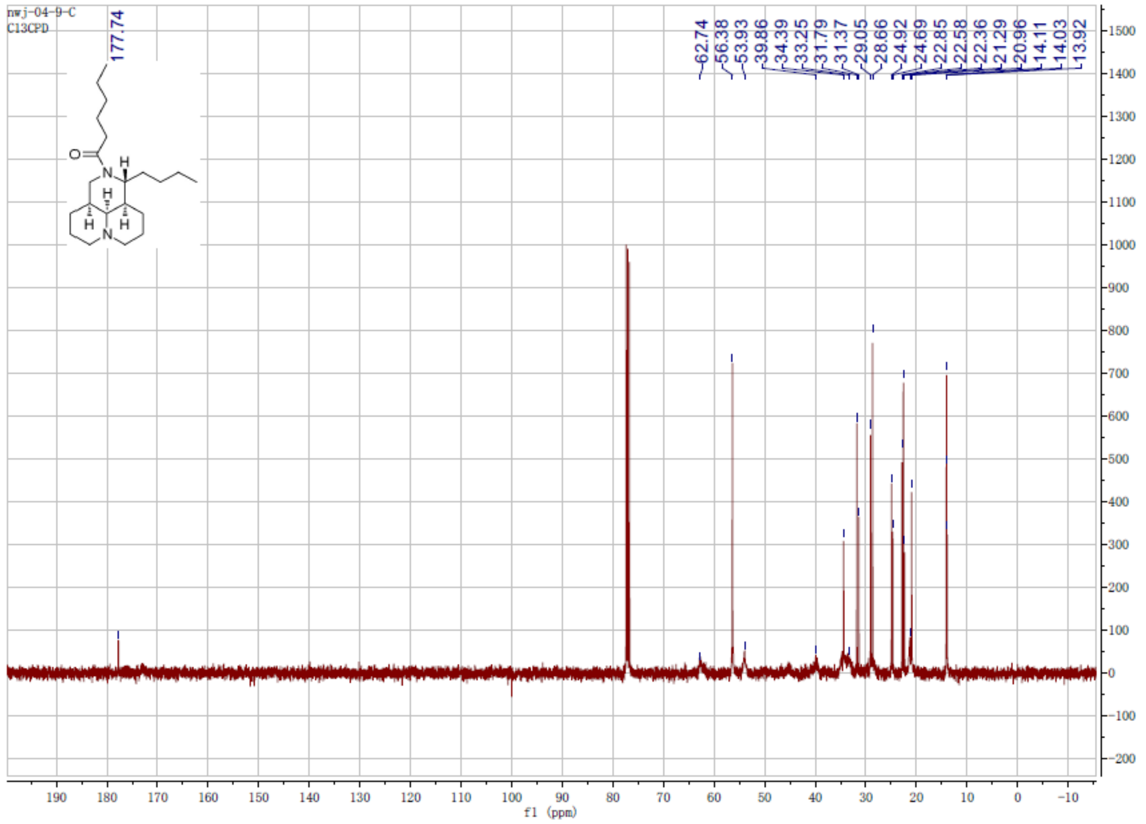

^{13}C NMR of compound 12

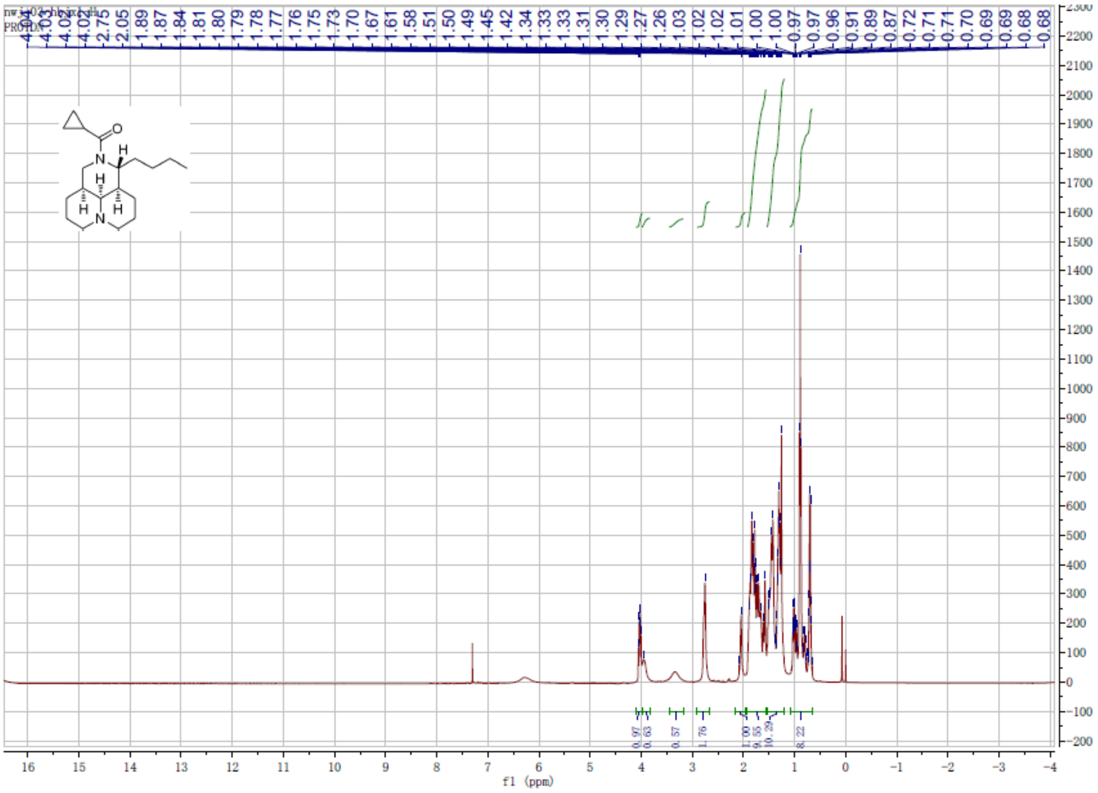

^{1}H NMR of compound 13

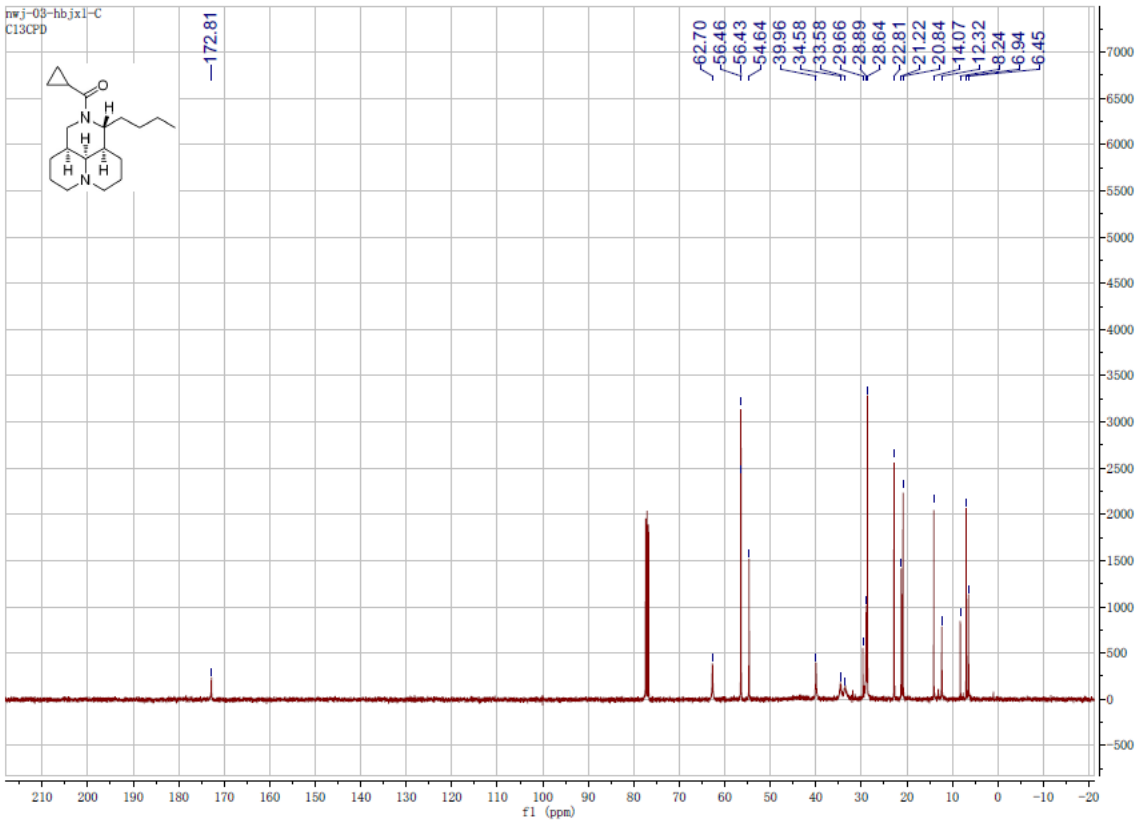

^{13}C NMR of compound 13

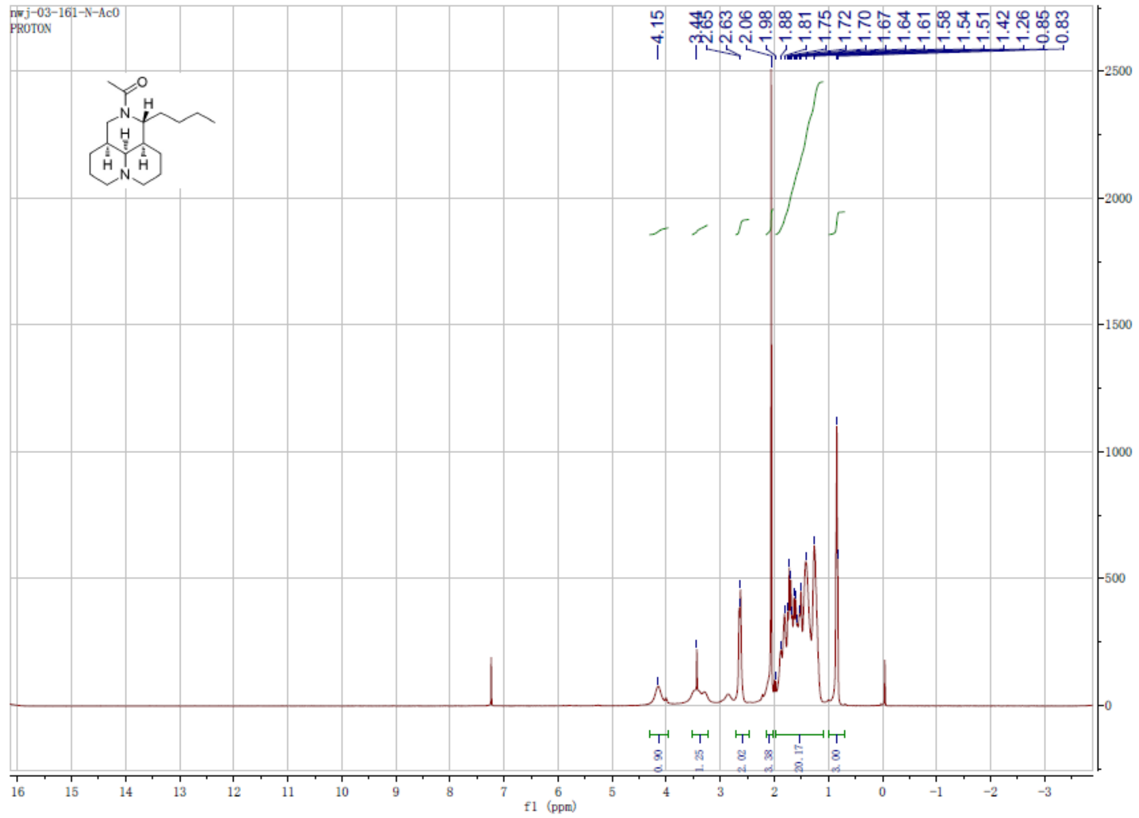

^{1}H NMR of compound 14

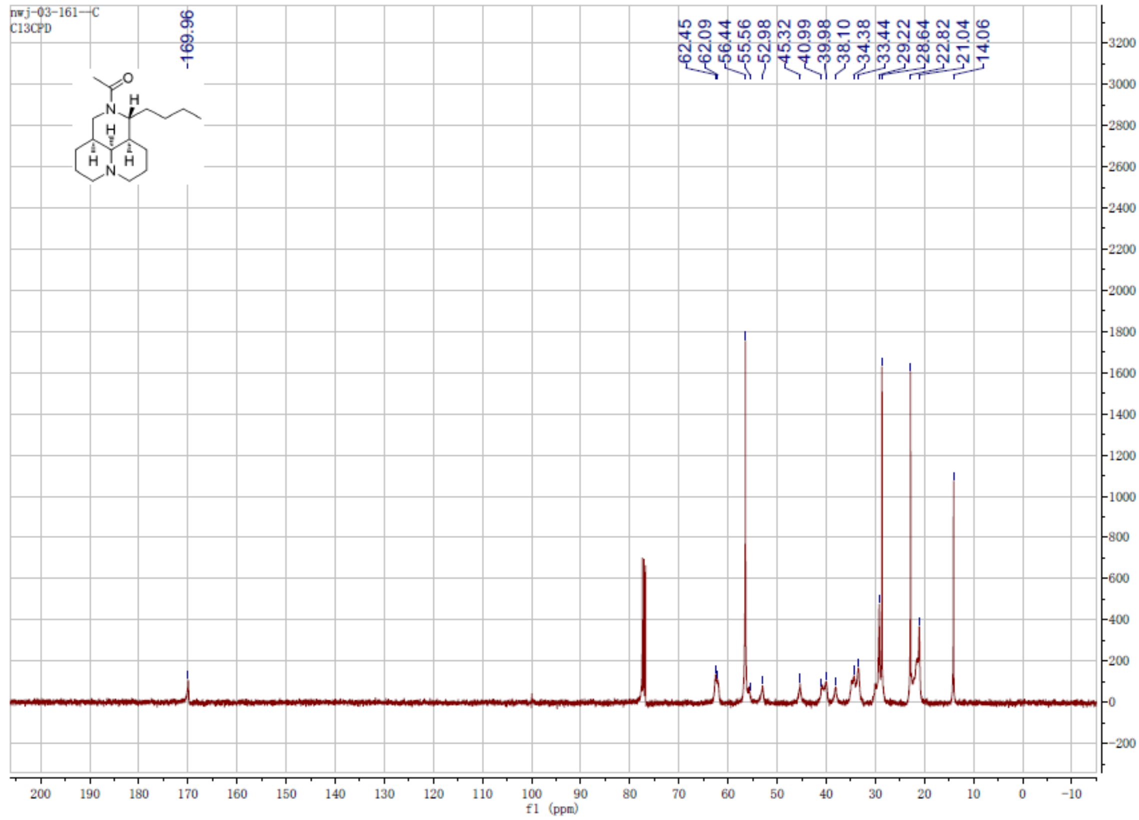

^{13}C NMR of compound 14

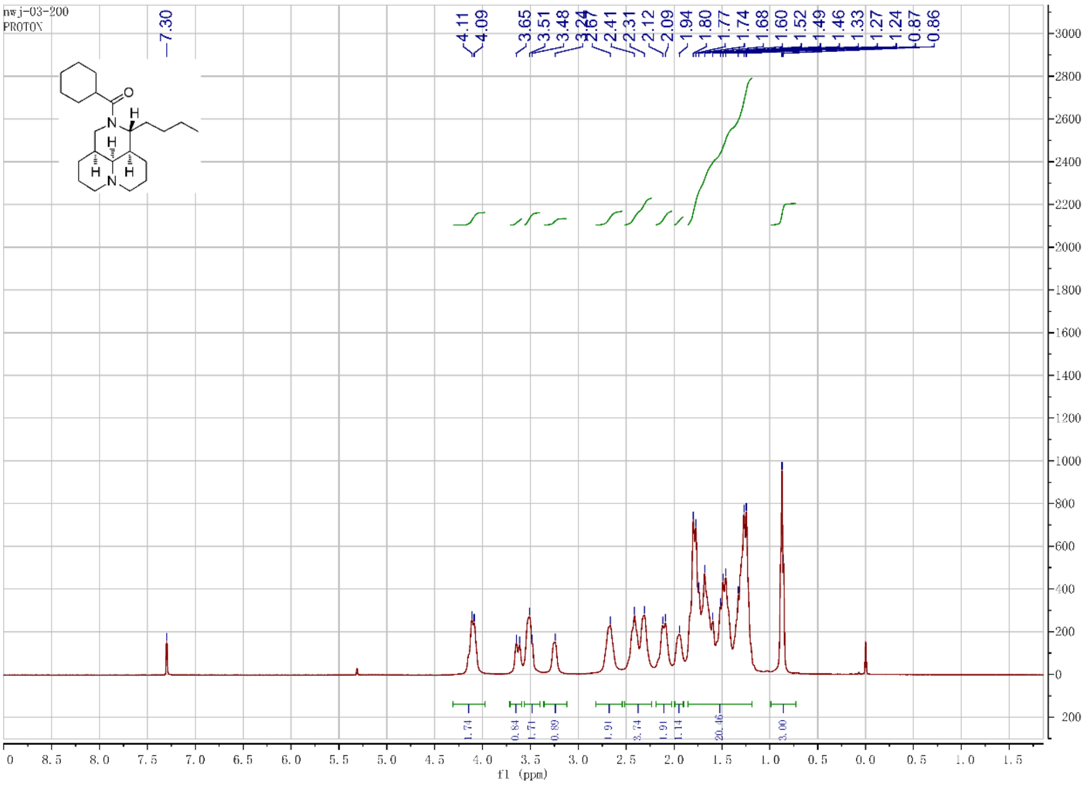

^{1}H NMR of compound 15

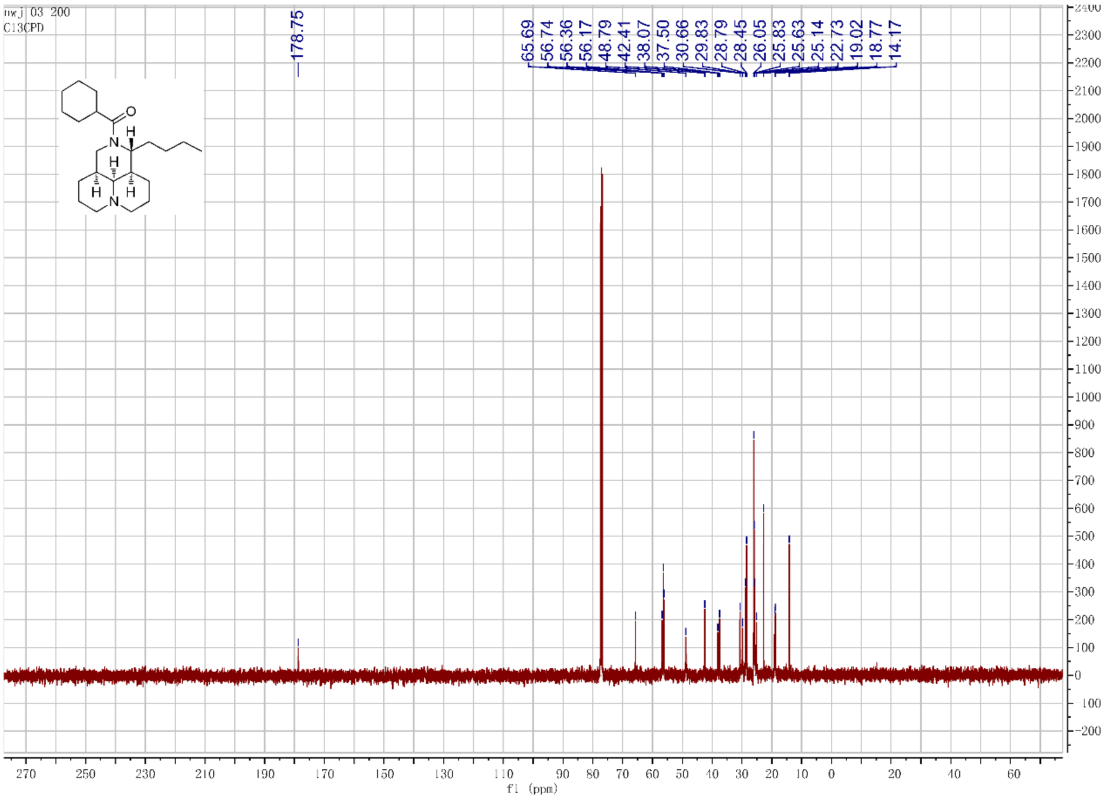

^{13}C NMR of compound 15

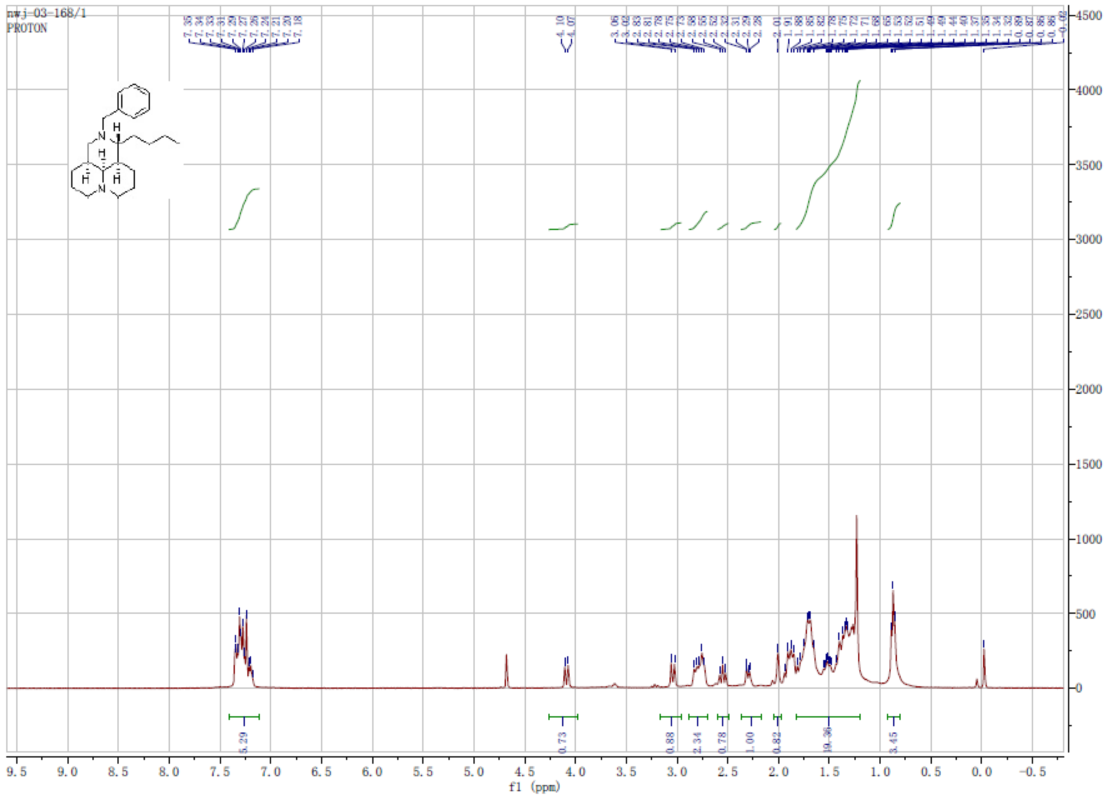

^{1}H NMR of compound 16

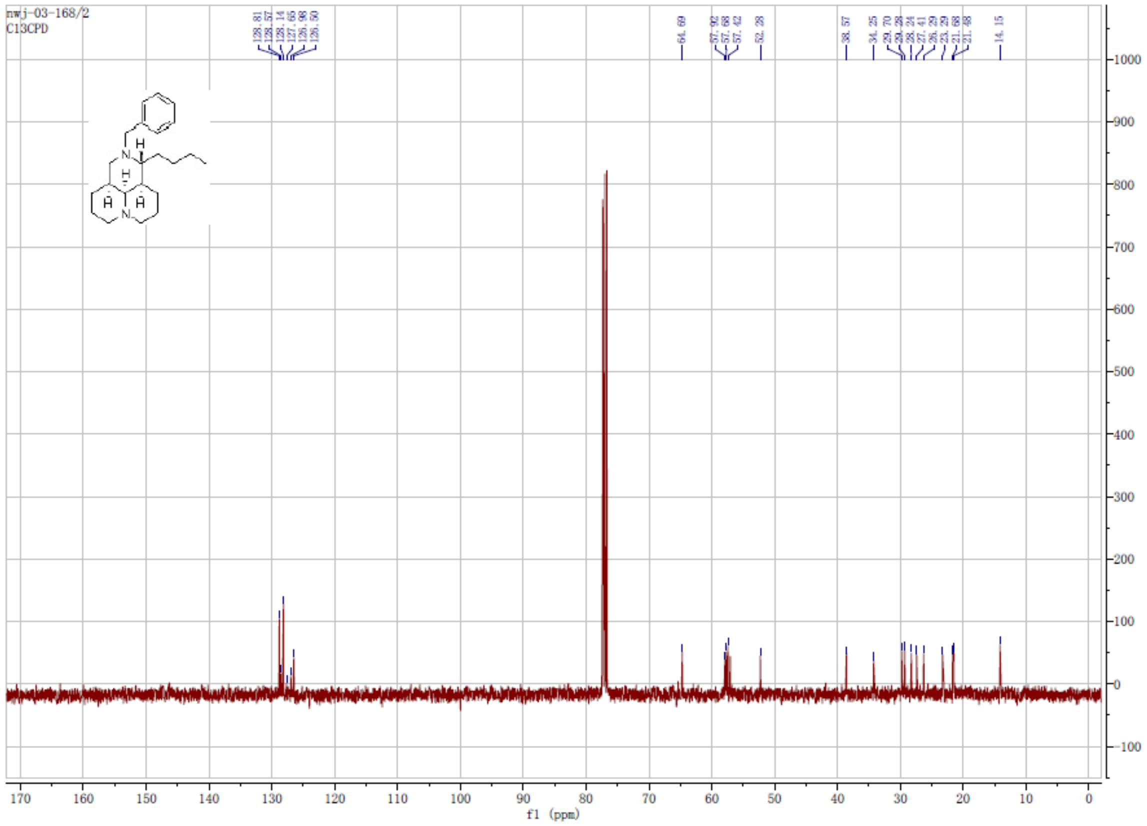

^{13}C NMR of compound 16

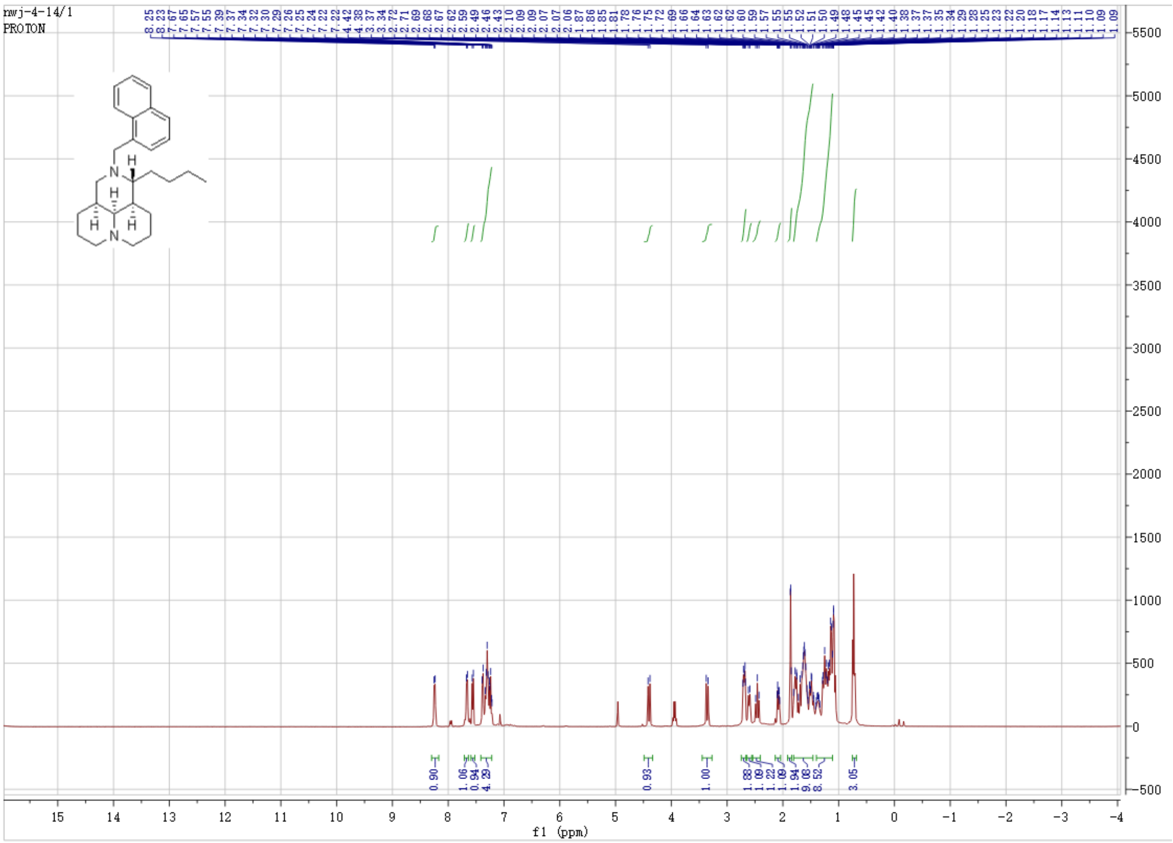

^{1}H NMR of compound 17

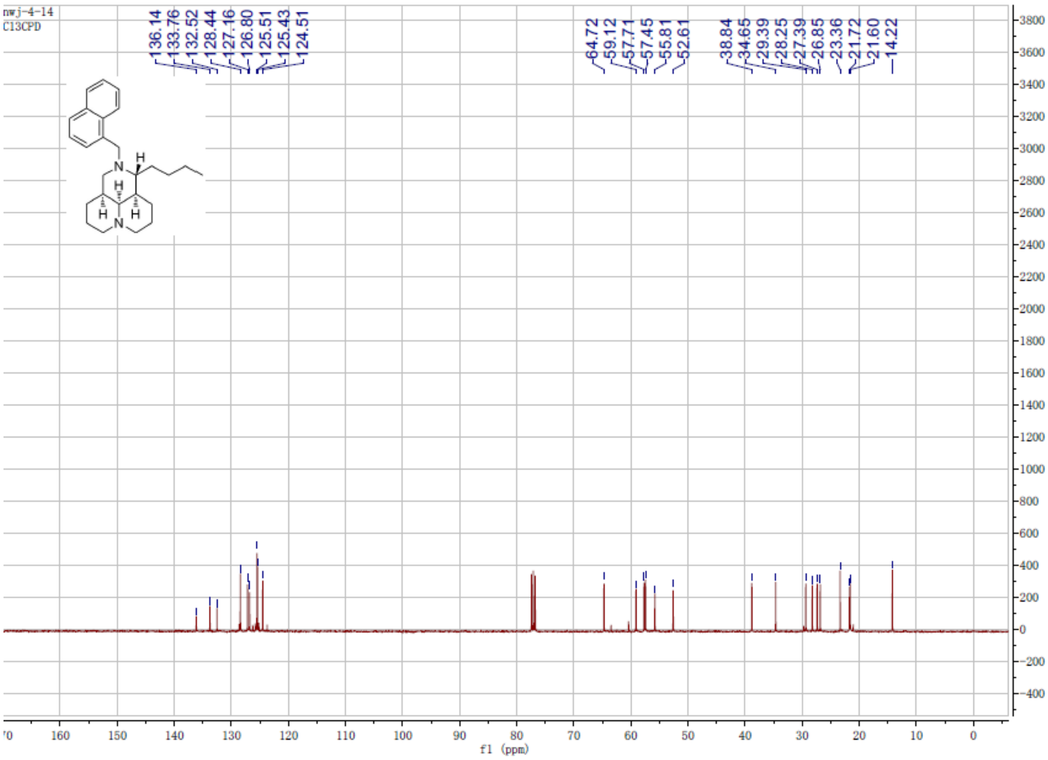

^{13}C NMR of compound 17

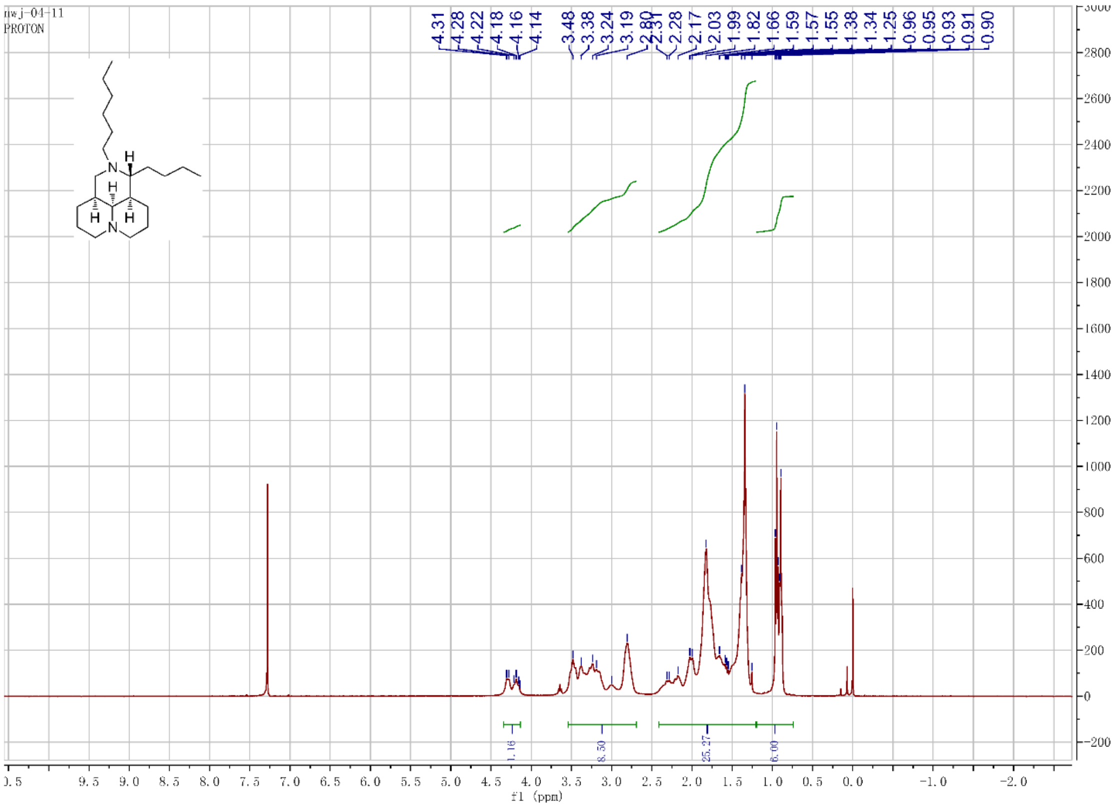

^{1}H NMR of compound 18

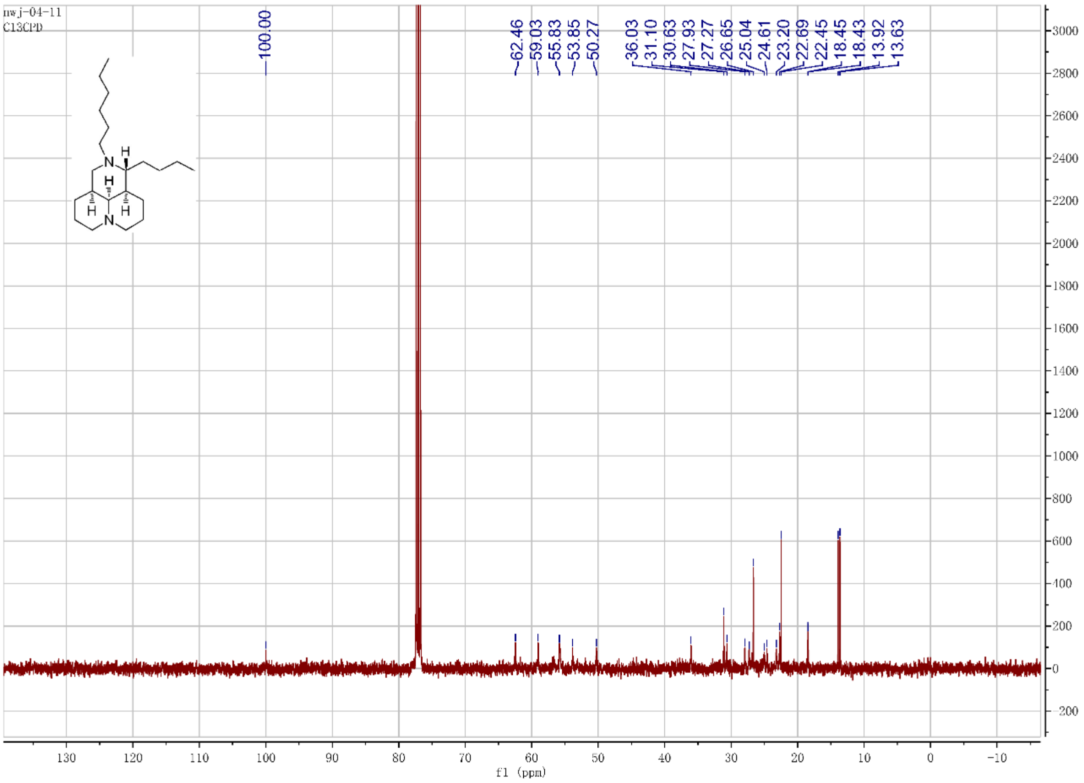

^{13}C NMR of compound 18

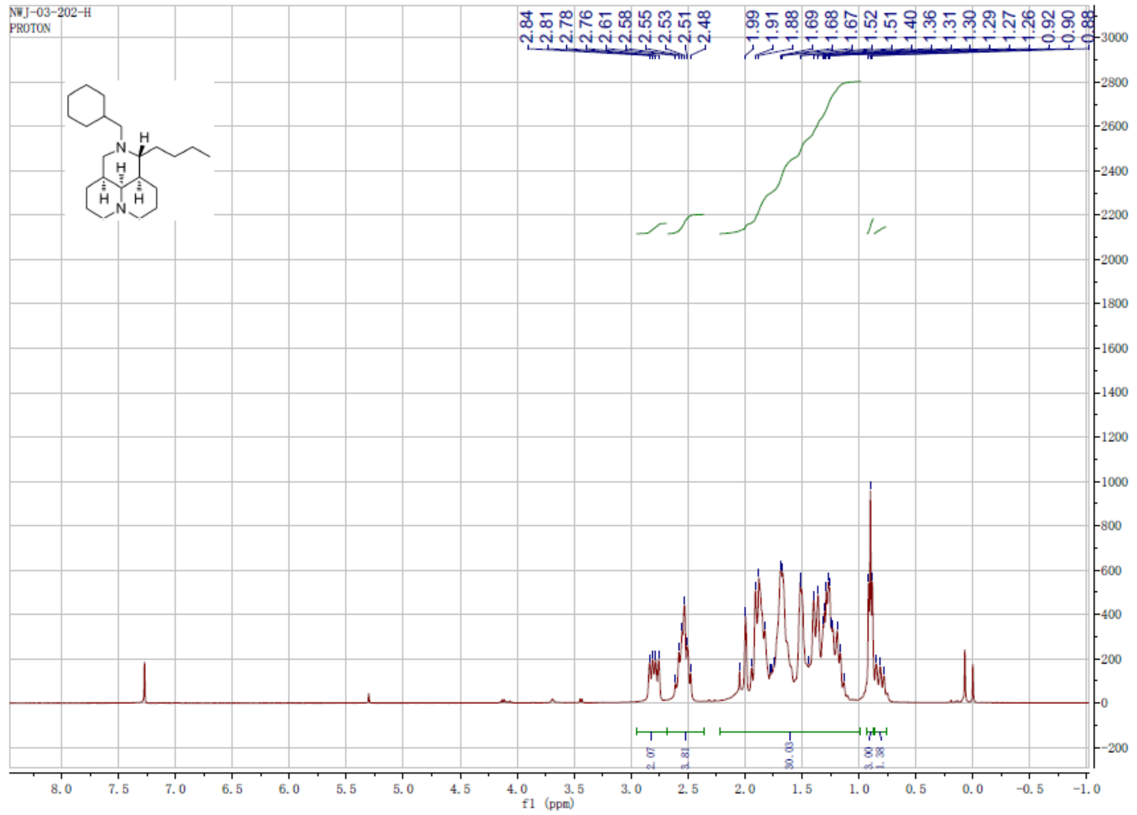

^{1}H NMR of compound 19

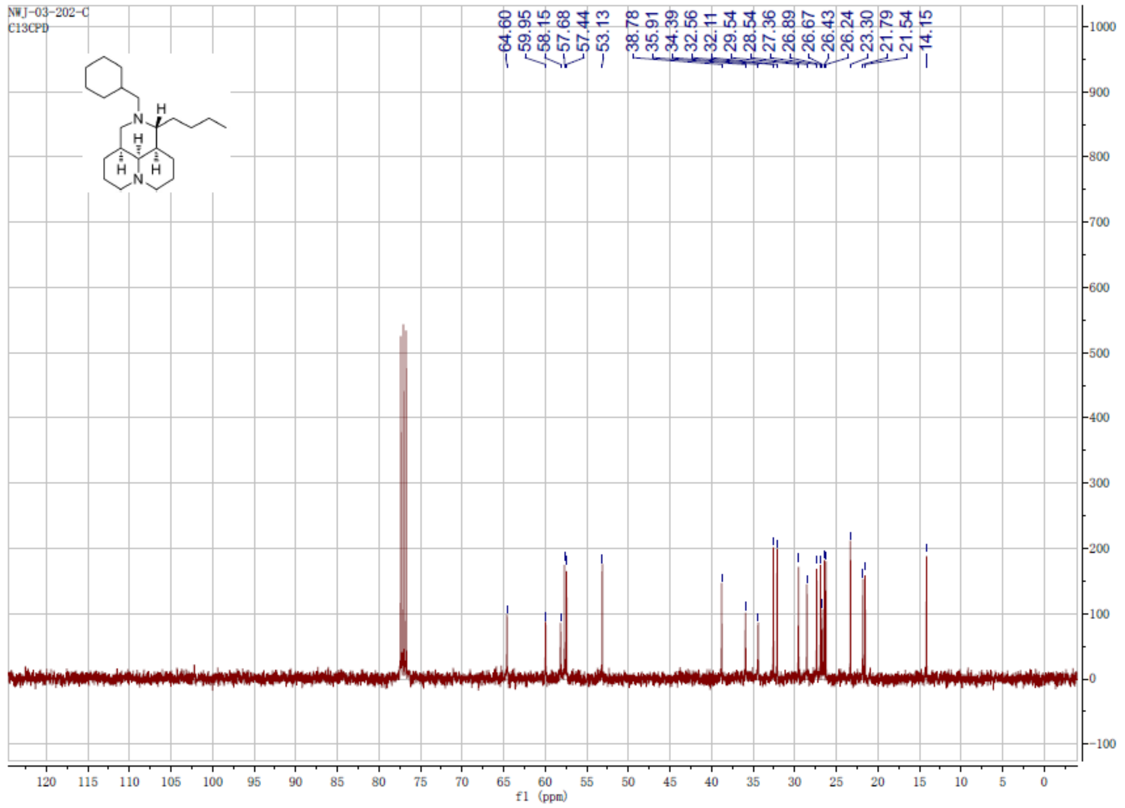

^{13}C NMR of compound 19

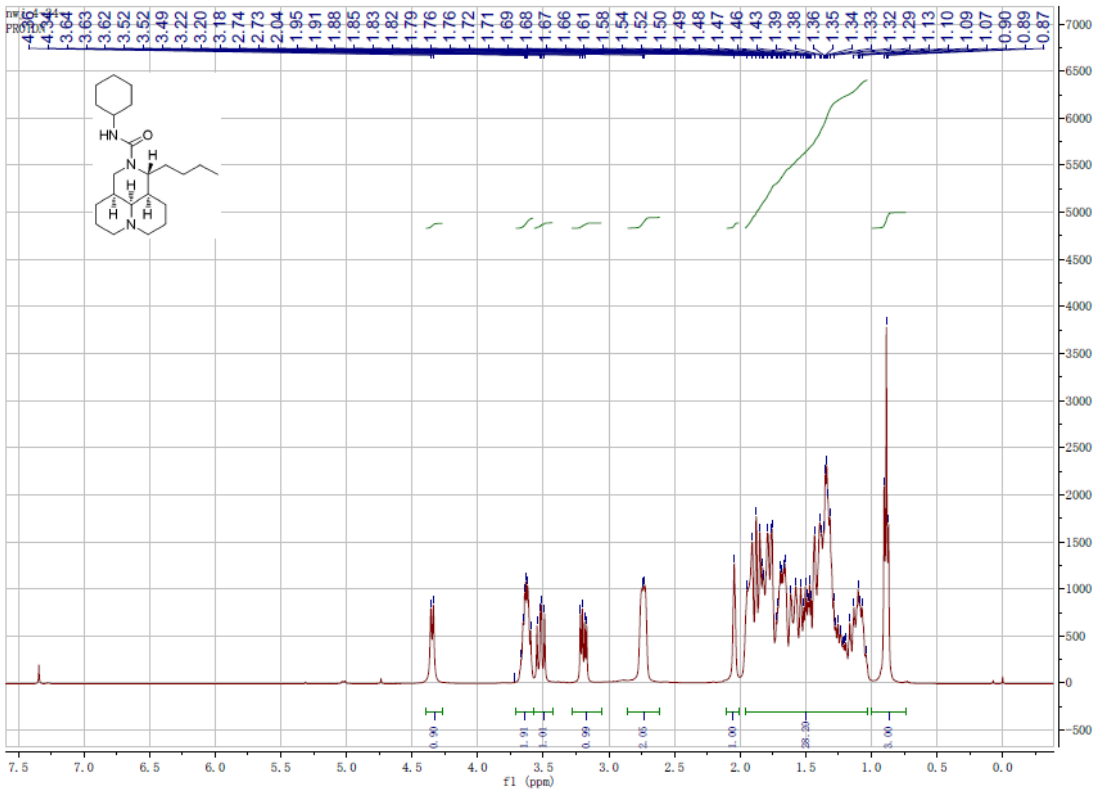

^{1}H NMR of compound 20

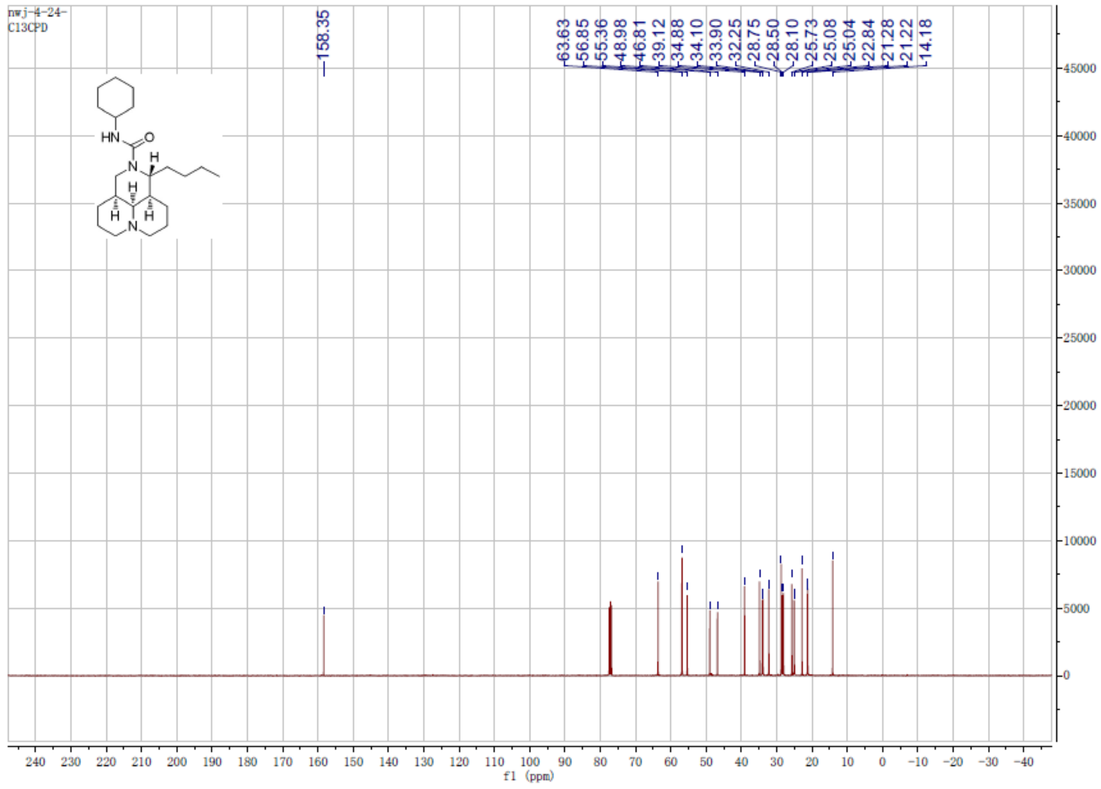

[1]C NMR of compound 20

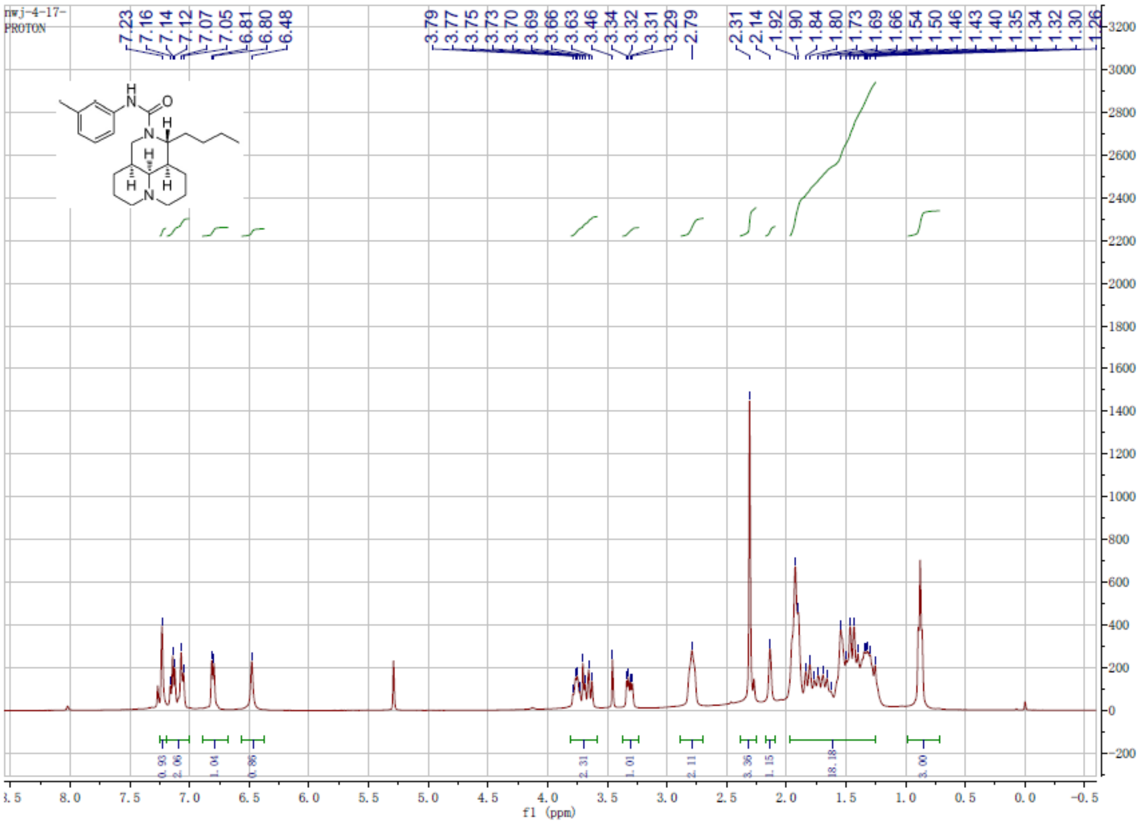

^{1}H NMR of compound 21

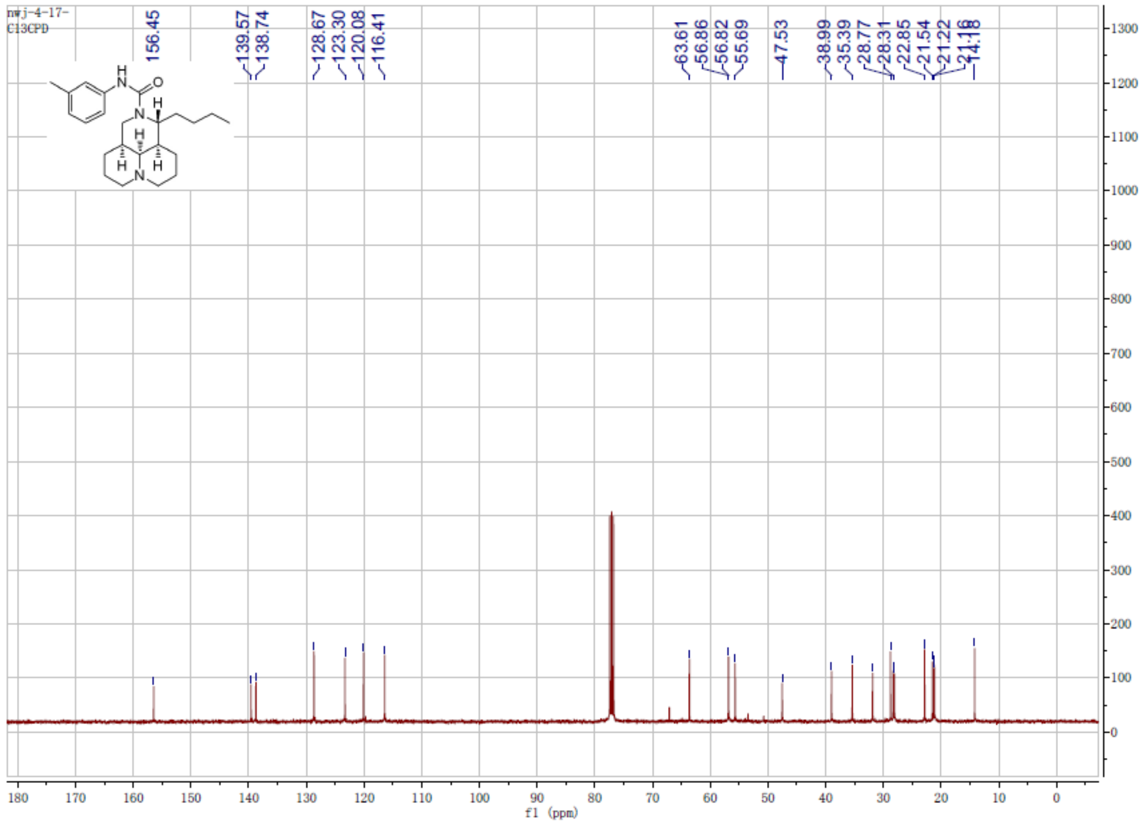

^{13}C NMR of compound 21

3. Detailed bioassay procedures

Detailed bioassay procedures for the anti-TMV activities

Compound solution preparation: Test compound was dissolved within a suitable amount of *N*,*N*-dimethylformamide and diluted with water containing 0.1% TW-80 to make a concentration of 500 μg/mL, and the aqueous solution was diluted to 100 μg/mL.

TMV preparation: Using Gooding's method (Gooding, G. V., Jr.; Hebert, T. T. A simple technique for purification of tobacco mosaic virus in large quantities. *Phytopathology* **1967**, *57*, 1285–1290), the upper leaves of *Nicotiana tabacum* L. inoculated with TMV were selected and ground in phosphate buffer and then filtered through double-layer pledget. The filtrate was centrifuged at 10000 g, treated with PEG twice, and centrifuged again. The whole experiment was processed at 4 °C. Absorbance value was estimated at 260 nm by ultraviolet spectrophotometer.

$$\text{Virus concentration} = (A_{260} \times \text{dilution ratio})/E_{1cm}^{0.1\%,260nm}$$

Antiviral Activity of Compounds against TMV in Vitro. Fresh leaf of the 5−6 growth stage of tobacco (N. tabacum L.) inoculated by the juice-leaf rubbing method (concentration of TMV was 6×10^{-3} μg/mL) was cut into halves along the main vein. The halves were immersed into the solution of test compounds and solvent (double-distilled water containing 0.1% TW-80) for 30 min, respectively, and then cultured at 25 °C for 72 h. The local lesion numbers were then counted.

Inactivation Effect of Compounds against TMV in Vivo. The virus was inhibited by mixing with the compound solution at the same volume for 30 min. The mixture was then inoculated on the growing leaves of the same ages, whereas another pot was inoculated with the mixture of solvent and the virus for control. The local lesion numbers were recorded 3−4 days after inoculation. There were three replicates for each compound.

Curative Effect of Compounds against TMV in Vivo. TMV (concentration of 6.0×10^{-3} mg/mL) was inoculated on the growing leaves of N. tabacum L. of the same ages. Then, the leaves were washed with water and dried. The compound solution was smeared on the leaves, whereas another pot was smeared with solvent for control. The local lesion numbers were then counted and recorded 3−4 days after inoculation. There were three replicates for each compound.

Protective Effect of Compounds against TMV in Vivo. The compound solution was smeared

on the growing N. tabacum L. leaves of the same ages. Another pot was smeared with solvent for control. After 12 h, the leaves were inoculated by the juice-leaf rubbing method and then washed with water. The local lesion numbers appearing 3−4 days after inoculation were counted. There were three replicates for each compound.

The in vitro and in vivo inhibition rates of the compound were then calculated according to the following formula (“av” means average, and controls were not treated with compound):

inhibition rate (%) = [(av local lesion number of control − av local lesion number of drugtreated)/av local lesion number of control] × 100%.

Detailed bioassay procedures for the insecticidal activities

Larvicidal Activities against oriental armyworm (Mythimna separata), diamondback moth (Plutella xylostella), cotton bollworm (Helicoverpa armigera), and corn borer (Ostrinia nubilalis): Stock solutions of each test compound was prepared in dimethylformamide at a concentration of 600 mg/L and then diluted to the required concentration (200, 100, and 50 mg/L) with water containing TW-20.

Leaf-dip method was used. Leaf discs (5 cm × 3 cm) were cut from fresh cabbage leaves (or other leaves) and then dipped into the test solution for 3 s. After air-drying, the treated leaf discs were placed individually into vertical tube (or Petri dishes) and the discs were infested with 10 larvae (for example: 10 second-instar diamondback moth larvae, 10 fourth-instar oriental armyworm larvae). Percentage mortalities were evaluated 3 days after treatment.

Evaluations were based on a percentage scale of 0–100, where 0 equals no activity and 100 equals total kill. Each treatment was performed at least two times.

Larvicidal Activities against Mosquito (Culex pipiens pallens). Twenty fourth-instar mosquito larvae were put into the 10 mL of the test solution (concentration 10, 5, and 2 mg/L). Percentage mortalities were evaluated 8 days after treatment.

Evaluations were based on a percentage scale of 0–100, where 0 equals no activity and 100 equals total kill. Each treatment was performed at least two times.

Aphicidal activity against aphid (Aphis Medicnginis Koch): About 60 aphids were transferred to the shoot with 3-5 fresh leaves of horsebean. The shoot with aphids was cut and dipped into the

solution of 600 μg/mL of test compound for 2 s, after removing extra solutions on the leaf; the aphids were raised in the shoot for 1 day. Percentage mortalities were evaluated by roughly counting the number of live and dead aphids. Evaluations were based on a percentage scale of 0–100, where 0 equals no activity and 100 equals total kill, and mortality rates were corrected using Abbott's formula. There were three replicates for each compound.

Acaricidal activity against adult spider mite (Tetranychus cinnabarinus): Sieva bean plants (*Phaseolus vulgaris* L.) when its primary leaves expanded to 10 cm were selected and cut back to one plant per pot. Adult mites were moved using a fine brush from the main colony to leaves of the test plants; each leaf was let to have about 60−100 adults. The mite-infested leaves were dipped into the test solution for 3 s with agitation, excess liquid was got rid of by shaking, and then the leaves were placed in a tube (10 cm inner diameter) lined with a piece of filter paper. Percentage mortalities were evaluated 4 days after test by roughly counting the number of live and dead adults. Evaluations were based on a percentage scale of 0–100, where 0 equals no activity and 100 equals total kill, and mortality rates were corrected using Abbott's formula. Each treatment was performed three times.

Detailed bioassay procedures for the fungicidal activities

The compounds were evaluated in mycelial growth tests in artificial media against 14 plant pathogens at rate of 50 mg/L.

Test compound was dissolved within a suitable amount of acetone and diluted with water containing 0.1% TW-80 to the concentration of 500 mg/L. To each petri dish was added 1 mL such solution and 9 mL culture medium to make a 50 mg/L of medicated tablet, whereas to another petri dish was added 1 mL sterilized water and 9 mL culture medium as blank control. A diameter of 4 mm of hyphae was cut by a hole puncher along the hyphae for bacteria to the outer plate and moved to the medicated tablet. Each treatment was performed three times. The dishes were stored in controlled environment cabinets (24±1°C) for 48 h, after which the diameter of mycelia growth was investigated and percentage inhibition was calculated.

Percentage inhibition (%) = (averaged diameter of mycelia in blank controls − averaged diameter of mycelia in medicated tablets) / averaged diameter of mycelia in blank controls

Table S1. Larvicidal and Acaricidal Activities of Compounds **1-21**.[a]

Compd.	armyworm[a]	bollworm[a]	borer[a]	moth[a]	aphid[a]	mite[a]	mosquito larva[b]
matrine	35±5	25±5	40±10	20±10	0	0	40±0
**1**	10±10	5±5	40±10	30±10	0	0	40±10
**2**	15±5	15±5	10±0	0	0	0	20±0
**3**	50±0	30±10	20±10	50±0	0	0	20±0
**4**	0	5±5	80±0	60±0	0	0	20±0
**5**	5±5	10±0	20±0	75±5	0	40	0
**6**	0	0	20±10	50±10	0	0	20±10
**7**	25±5	20±0	0	60±0	30	0	30±10
**8**	0	0	40±10	40±10	0	0	0
**9**	0	0	10±10	45±5	0	0	0
**10**	0	10±10	10±0	70±0	0	0	0
**11**	0	0	20±10	75±5	0	40	0
**12**	20±10	10±10	0	40±10	0	0	0
**13**	5±5	5±5	40±0	65±5	0	0	0
**14**	15±5	15±5	30±0	0	0	0	0
**15**	0	0	30±0	60±0	0	0	20±10
**16**	10±10	15±5	0	60±0	0	20	0
**17**	25±5	25±5	30±10	60±10	0	0	30±0
**18**	20±10	15±5	40±0	70±0	0	0	60±0
**19**	80±0	70±0	40±0	70±0	0	0	20±10
**20**	85±5	75±5	100±0/40±0[c]	85±5	0	0	40±0
**21**	35±5	25±5	100±0/20±10[c]	80±0	0	0	100/100[d]/20±10[e]

[a]Armyworm (oriental armyworm, *Mythimna Separate*), bollworm (cotton bollworm, *Helicoverpa Armigera*), borer (corn bborer, *Ostrinia Nubilalis*), moth (diamond back moth, *Plutella Xylostella*),

aphid (*Aphis Medicnginis* Koch), mite (*Tetranychus cinnabarinus*); Mortality (%) tested at 600 μg/mL unless otherwise stated. [b]Mosquito larva (*C. Pipiens Pallens*), mortality (%) tested at 10 μg/mL unless otherwise stated. [c] Tested at 200 μg/mL. [d]Tested at 5 μg/mL. [e]Tested at 2 μg/mL.

Table S2. Fungicidal Activity of Compounds **1-21** against Phytopathogens.

Compd	Fungicidal activity (%)/50 μg/mL													
	F.C.	*C.H.*	*P.P.*	*R.C.*	*B.M.*	*C.O.*	*F.M.*	*A.S.*	*F.G.*	*P.I.*	*P.C.*	*S.S.*	*B.C.*	*R.S.*
Chlorothalonil	73±1	81±2	74±1	96±2	96±2	96±1	83±2	97±1	35±2	97±1	98±1	97±1	94±2	96±1
Carbendazim	97±1	35±2	97±1	98±1	97±1	94±2	96±1	65±2	38±1	88±2	58±2	80±1	65±3	96±1
matrine	25±2	23±1	31±2	44±1	74±1	54±1	43±1	5±2	7±1	30±1	12±2	8±1	10±1	13±3
**1**	19±1	17±1	30±3	29±1	21±3	19±3	8±3	8±2	23±2	6±1	31±1	43±2	17±2	6±1
**2**	14±2	14±2	39±1	52±3	15±2	0±0	8±3	8±2	23±3	12±2	13±3	24±1	4±3	6±3
**3**	27±1	52±2	66±2	80±2	41±1	42±2	25±2	17±1	29±1	13±2	12±1	33±3	8±3	6±3
**4**	8±3	21±3	47±2	20±2	21±3	16±1	21±2	17±3	19±2	6±1	31±2	6±3	4±3	6±1
**5**	16±1	17±1	51±2	19±3	26±3	16±2	13±1	25±1	39±3	25±3	12±3	24±2	13±2	8±3
**6**	14±2	21±3	35±1	23±2	20±2	16±1	13±3	17±3	26±1	25±1	31±1	11±3	8±3	12±3
**7**	11±2	24±1	49±3	47±3	21±3	16±3	13±3	8±1	26±2	13±3	19±3	43±1	13±2	16±2
**8**	19±2	28±2	64±3	26±3	39±3	10±2	17±3	8±3	26±3	6±3	63±2	39±2	12±3	12±3
**9**	16±2	20±3	45±2	44±1	18±2	13±1	4±2	17±3	29±1	12±2	31±2	39±1	12±1	6±1
**10**	16±1	17±1	34±2	17±2	23±1	13±2	21±3	8±3	39±2	13±3	31±1	6±3	17±1	16±3
**11**	11±2	10±2	31±1	7±3	21±3	16±1	17±3	17±3	29±1	19±3	6±2	4±3	12±3	16±3
**12**	16±2	21±2	43±1	22±1	26±3	13±2	17±1	8±1	36±3	31±1	56±3	11±1	21±2	10±1
**13**	16±1	17±1	51±2	9±2	15±2	12±2	13±3	16±2	6±1	6±3	31±3	20±2	12±3	10±2
**14**	14±3	14±2	51±2	20±2	23±1	19±2	0±0	25±2	13±1	12±2	6±1	33±3	25±1	27±1

**15**	24±2	17±2	34±3	33±2	33±2	26±3	25±2	8±3	10±2	13±3	19±3	24±1	8±1	8±3
**16**	16±1	24±1	42±1	16±2	21±3	19±2	21±1	25±2	16±1	6±3	18±3	37±2	42±1	2±3
**17**	24±2	38±2	57±2	66±2	28±1	23±3	4±3	17±2	32±3	44±1	19±1	39±3	13±3	6±2
**18**	35±1	66±3	77±2	79±1	51±2	55±3	67±3	8±1	48±2	25±2	38±2	52±2	25±1	6±3
**19**	16±1	10±2	38±2	12±3	21±3	16±2	8±2	25±2	23±3	19±1	69±2	24±1	12±3	37±1
**20**	16±2	28±3	41±3	17±2	15±2	13±3	8±3	8±2	26±3	13±2	31±1	15±3	4±1	2±2
**21**	8±3	10±2	35±1	8±2	13±3	19±2	17±1	42±3	55±1	19±1	75±1	72±1	17±3	33±2

[a]*F.C., Fusarium oxysporum sp. cucumeris; C.H., Cercospora arachidicola Hori; P.P., Physalospora piricola; R.C., Rhizoctonia cerealis; B.M., Bipolaris maydis; C.O., Colletotrichum orbiculare; F.M., Fusarium moniliforme; A.S., Alternaria solani; F.G., Fusarium graminearum; P.I., Phytophthora infestans; P.C., Phytophthora capsici; S.S., Sclerotinia sclerotiorum; B.C., Botrytis cinerea. R.S., Rhizoctonia solani.*
